# Synergizing high-sugar traits and interspecific diversity: temporal dynamics and trade-offs in temperate grasslands

**DOI:** 10.3389/fpls.2026.1835923

**Published:** 2026-05-20

**Authors:** Fan Ye, Eun Joong Kim, Vincent Niderkorn, Nigel David Scollan, Fujiang Hou

**Affiliations:** 1 China-Kazakhstan Belt and Road Joint Laboratory on Grassland Ecological Restoration, Key Laboratory of Grassland Livestock Industry Innovation, Ministry of Agriculture and Rural Affairs, Engineering Technology Research Center for Ecological Restoration and Utilization of Degraded Grassland in Northwest China, National Forestry and Grassland Administration, College of Pastoral Agriculture Science and Technology, Lanzhou University, Lanzhou, Gansu, China; 2 Research Institute for Innovative Animal Science, Kyungpook National University, Daegu, Republic of Korea; 3 French National Institute for Agriculture, Food, and Environment, Paris, France; 4 Institute for Global Food Security, Queen’s University Belfast, Belfast, United Kingdom

**Keywords:** botanical composition, forage yield, high-sugar ryegrass, nutritive value, stability, white clover

## Abstract

**Introduction:**

Temperate grasslands are essential for sustainable ruminant production, yet their seasonal stability and nutritional balance are increasingly threatened by climate shifting. While high-sugar ryegrass (HSG) cultivars and grass-legume mixtures offer solutions to these constraints, their integrated performance across productivity, quality, and stability dimensions requires holistic evaluation.

**Methods:**

This study conducted a field experiment in Aberystwyth, UK, employing a two-factor design with five grass components (three HSG cultivars, a tri-mixture, and a standard control) and two sowing modes (pure grass vs. white clover mixtures). Across nine harvests, we assessed temporal dynamics in dry matter (DM) yield, community composition, and nutritive value. Additionally, the Coupling Coordination Degree, food equivalent unit (FEU), and food equivalent unit productivity (PFEU) were comprehensively evaluated as independent indices to quantify trade-offs and synergies.

**Results:**

The pure-sown HSG cultivar AberMagic (AM) maximized early-season DM yield (9144.48 kg·ha^-1^) but experienced significant mid-season declines. Conversely, grass-clover mixtures mitigated this “summer slump” via temporal niche differentiation, significantly improving yield stability and biological weed suppression (grass weeds< 0.5%). Nutritional analysis revealed a physiological trade-off: pure HSG stands delivered superior energy density through elevated water-soluble carbohydrates (WSC), whereas mixtures provided a more balanced nutritional profile, significantly increasing late-season crude protein (CP) to 22.20%–28.05% and reducing neutral detergent fiber (NDF).

**Discussion:**

Management models must align with specific agronomic goals. Pure AM is the premier choice for intensive systems prioritizing maximum total output. For long-term grazing requiring a stable feed supply, the AberAvon (AA) and white clover (WC) mixture is optimal due to its exceptional late-season recovery and excellent synergistic performance across indicators. Furthermore, the consistent underperformance of standard varieties underscores the absolute necessity of utilizing improved traits. Synergizing the ecological resilience of multi-species mixtures with the improved traits establishes an optimal framework for sustainable and highly coordinated forage systems.

## 1 Introduction

High-sugar grasses play a critical role in underpinning sustainable ruminant production by offering a cost-effective and energy-dense nutrient base (Edwards et al., 2007). As a representative high-sugar species, perennial ryegrass (*Lolium perenne* L.) locally dominates temperate grasslands due to its substantial annual dry matter yields (typically ranging from 10 to 15 t/ha) and exceptional dry matter digestibility (often exceeding 80%) (Wilkins and Humphreys, 2003). However, the reliability of such monoculture swards is increasingly vulnerable to shifting climate patterns. Studies by Isbell et al. (2015) and Hofer et al. (2016) demonstrate that compared to diverse plant communities, monocultures are disproportionately affected by climate extremes such as droughts and heatwaves due to a lack of functional redundancy and interspecific buffering. Consequently, driven by elevated summer temperatures and progressive soil nitrogen depletion, pure stands of cool-season grasses often suffer severe seasonal yield and quality declines (Wang et al., 2015). Under increased temperature, the chlorophyll content of creeping bentgrass (*Agrostis stolonifera* L.) decreases by 7%–20% (Huang et al., 1998), the non-structural carbohydrate content of Kentucky bluegrass (*Poa pratensis* L.) decreases by 23-64% (Watschke et al., 1970), and the peroxidase activity of perennial ryegrass decreases by more than 50% (Xu et al., 2006). Such seasonal deficits ultimately constrain grazing efficiency and force reliance on costly feed supplements (Weinert-Nelson et al., 2021). Therefore, developing grassland management strategies that simultaneously maintain high productivity, nutritional balance, and seasonal stability is a priority goal for modern animal husbandry.

One pathway to enhancing forage utilization efficiency is through breeding high-sugar ryegrass (HSG) cultivars, designed to elevate water-soluble carbohydrate (WSC) concentrations and improve the energy-to-protein balance in ruminants (Parsons et al., 2011). While HSG cultivars demonstrate superior production performance and high-sugar traits, their monocultures often lack the resilience to withstand environmental stress and weed invasion (Crews et al., 2018). Integrating leguminous species into the grass matrix offers a functional solution to these stability constraints (Jaramillo et al., 2021). Drawing on “Compensatory effects”, multi-species swards can buffer seasonal production dips through temporal niche partitioning and biological nitrogen fixation (Liang et al., 2022), effectively marrying improved trait with community-level ecological resilience.

While pure HSG stands and grass-legume mixtures may each possess distinct advantages (Sollenberger and Dubeux, 2022), few studies have evaluated their temporal dynamics and trade-offs across multiple dimensions. For instance, a treatment that maximizes early-season yield might suffer from severe mid-season declines or nutritional imbalances (Fontaneli et al., 2001). Traditional evaluations often focus on single dimensions such as total annual yield or specific nutrient fractions, thereby failing to address the holistic and seasonal performance of the sward. Therefore, there is a need for a comprehensive assessment utilizing independent evaluation indices including the coupling coordination degree, food equivalent unit (FEU), and food equivalent unit productivity (PFEU) to quantify how high-sugar traits interact with interspecific diversity to affect productivity, nutritive value, and ecosystem sustainability simultaneously.

To address these knowledge gaps, rather than relying on a single integrated model, independent evaluation indices were comprehensively employed to assess the trade-offs and synergies across dimensions among treatments. The specific objectives were: (i) to characterize the seasonal dynamics of dry matter (DM) yield and determine whether temporal niche differentiation in mixtures can mitigate the summer production decline; (ii) to analyze the seasonal dynamics of sward components and nutritive value, specifically exploring the physiological trade-offs between energy and protein; and (iii) to evaluate the coupling coordination degree, FEU, and PFEU to identify optimal management models and validate the necessity of utilizing improved traits for sustainable forage systems.

## 2 Materials and methods

### 2.1 Experimental site and overview

**Figure 1:**
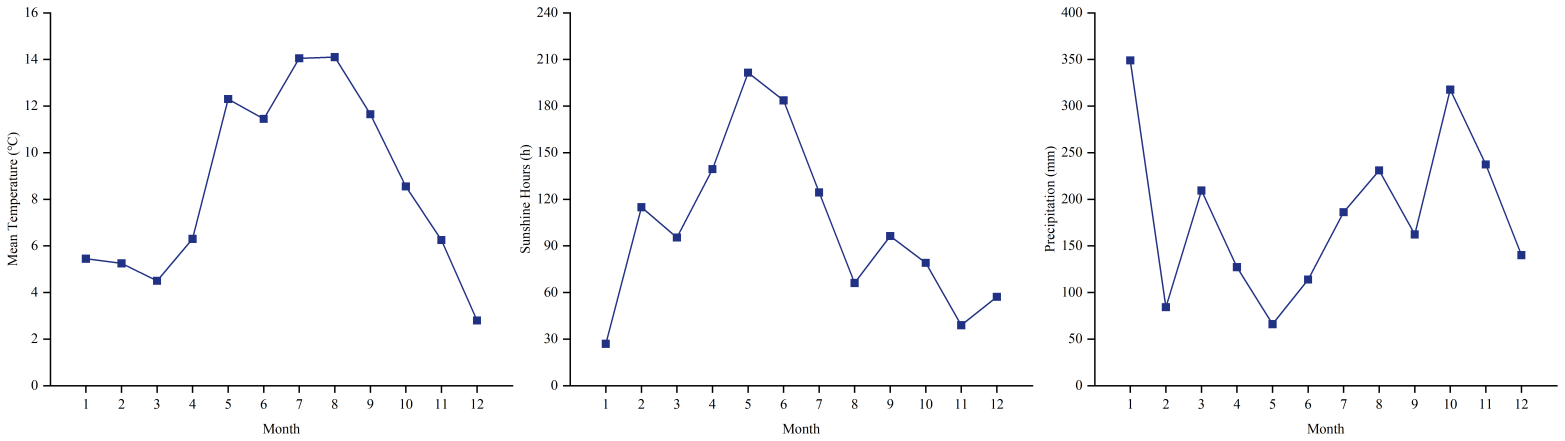
Monthly mean temperature, sunshine hours, and precipitation in Aberystwyth in 2008.

The field experiment was conducted in 2008 at the experimental station of Aberystwyth University, UK. The station is located on the west coast of central Wales (52°25’ N, 4°03’ W) at an elevation of 7m above sea level. According to the Köppen climate classification, the region is categorized as Cfb (temperate oceanic climate) (Cui et al., 2021), characterized by mild conditions with warm, humid summers and evenly distributed rainfall throughout the year. **Figure 1** illustrates the monthly mean temperature, sunshine duration, and precipitation during the experimental period (2008). The year 2008 was generally wet, with a total annual precipitation of 2223.8mm. During the harvest season (April to October), the total precipitation was 1204mm, and the mean temperature was 11.2°C (Met Office, 2012).

### 2.2 Experimental design

The experiment followed a randomized block design with 10 treatments (**Table 1**), formed by a two-factor design of five grass components and two sowing modes, with four replicates. The five grass components included three high-sugar perennial ryegrass (*Lolium perenne* L.) cultivars (AberAvon (AA), AberMagic (AM), and AberStar (AS)), a ‘Tri-mixture’ (Tri) (comprising equal proportions of these three cultivars), and a standard variety ‘Premium’ (PR) as the control. The two sowing modes were defined as pure grass (sown without legumes) and grass-legume mixtures (sown with white clover (WC), *Trifolium repens* L.).

**Table 1:** Experimental treatments and abbreviations.

Grass component	Sowing mode	Abbreviation
AberAvon	Pure Grass	AA
AberMagic	Pure Grass	AM
AberStar	Pure Grass	AS
Tri-mixture	Pure Grass	Tri
Premium	Pure Grass	PR
AberAvon	+White clover	AA+WC
AberMagic	+White clover	AM+WC
AberStar	+White clover	AS+WC
Tri-mixture	+White clover	Tri+WC
Premium	+White clover	PR+WC

### 2.3 Field management

Establishment and sowing: The experimental site was sown on 9 September 2007. Prior to sowing, the soil was prepared by rotary tillage to a depth of 30cm. All seeds were supplied by the Institute of Biological, Environmental & Rural Sciences (IBERS), UK. Sowing was performed by broadcasting. The specific seeding rates are detailed in **Table 2**, calculated using the following formula (Dai et al., 2013):

**Table 2:** Sowing rates of high-sugar perennial ryegrass cultivars and white clover in pure grass stands and their mixtures.

Treatment	Ryegrass seeding rate (kg·ha^-1^)				White clover seeding rate (kg·ha^-1^)
	AberAvon	AberMagic	AberStar	Premium	
AA	31.69	0	0	0	0
AM	0	31.69	0	0	0
AS	0	0	31.69	0	0
Tri	10.56	10.56	10.56	0	0
PR	0	0	0	31.69	0
AA+WC	22.18	0	0	0	3.09
AM+WC	0	22.18	0	0	3.09
AS+WC	0	0	22.18	0	3.09
Tri+WC	7.39	7.39	7.39	0	3.09
PR+WC	0	0	0	22.18	3.09


SR=(TD×R×10,000)/(SPK×P×G)

where *SR* is the actual seeding rate (kg·ha^-1^), *TD* is the total target density, set at 1200 seeds·m^-2^, *R* is the seeding proportion (100% for ryegrass in monocultures; 70% for ryegrass and 30% for white clover in mixtures); *SPK* represents the number of seeds per kilogram (420,000 for ryegrass and 1,400,000 for white clover); *P* denotes purity (taken as 98% for both species); and *G* indicates the germination percentage, set at 92% for ryegrass and 85% for white clover.

Fertilization management: Before sowing, plots received a basal fertilizer application (NPK 15-15-15) at 150 kg·ha^-1^. Following each harvest, a compound fertilizer (NPK 21-6-13) was top-dressed at a rate of 40 kg·ha^-1^ (Lobley et al., 2016).

Mowing regime: An initial uniform mowing was carried out on all plots on 26 March 2008. Subsequently, mowing was conducted at 21-day intervals to simulate a rotational grazing regime (Grace et al., 2018), resulting in a total of 9 mowings.

### 2.4 Sampling and measurements

Biomass sampling: At each mowing event, a 1 m×1 m quadrat was randomly positioned within each plot. Above-ground biomass was harvested within the quadrat at a stubble height of 5cm (Vinther, 2006).

Productivity and botanical composition: The harvested biomass was manually separated into four components: perennial ryegrass (PRG), white clover (WC), broad-leaved weeds (BLW), and grassy weeds (GW). The fresh and dry weights of each component were recorded to calculate their specific proportions in the total community biomass. Total DM yield was calculated by summing the dry weights of these four components.

Stability: The temporal coefficient of variation (CV) based on data from the nine harvests was calculated for each treatment to assess yield stability (Lehman and Tilman, 2000).

Nutritive value: Dried samples from each harvest were ground and analyzed using near-infrared reflectance spectroscopy (NIRS) to determine dry matter digestibility (DMD), neutral detergent fiber (NDF), acid detergent fiber (ADF), water-soluble carbohydrates (WSC), and crude protein (CP) (Stuth et al., 2003). Ether extract (EE) was determined using the Soxhlet extraction method, and crude ash (Ash) content was measured by calcination in a muffle furnace at 550°C.

### 2.5 Statistical analysis

Preliminary data processing and summarization were performed using Microsoft Excel. Statistical analyses were conducted using SPSS Statistics 27. Analysis of variance (ANOVA) was employed to evaluate the significance of differences among treatments and harvest times, followed by Duncan’s multiple range test for *post-hoc* multiple comparisons (P< 0.05). The relationships among variables were assessed using Pearson correlation coefficients visualized via a correlation heatmap. To model the temporal dynamics of forage yield and nutritive value across the growing season, regression analyses (including linear, polynomial, and exponential models) were performed, with optimal models selected based on the coefficient of determination (*R*^2^). All figures, curve fitting, and parameter estimations were generated using Origin 2024.

### 2.6 Comprehensive evaluation

Dry matter intake (DMI), expressed as a percentage of ruminant body weight, was calculated based on the NDF content according to the following formula:


DMI=120/NDF

Relative feed value (RFV) is an internationally recognized index for forage quality assessment that integrates both digestibility and potential dry matter intake (Yang et al., 2022). The formula is as follows:


RFV=DMI×DMD/1.29

Food equivalent unit (FEU) is a comprehensive evaluation unit based on energy and protein (Jiang et al., 2024). The calculation is as follows:

1. Nitrogen-free extract (NFE) was calculated using the following formula:


NFE=100−CP−EE−NDF−Ash

2. Energy (H) was calculated using the following formula:


H=(5.72×CP+9.5×EE+4.79×ADF+4.03×NFE)×4.182/100

3. Food Equivalent Unit (FEU) was calculated using the following formula:


FEU=DMD×(H×0.042+CP×0.0033)/100

Food equivalent unit productivity (PFEU) is defined as the food equivalent units of forage dry matter per unit area, calculated as follows:


$$PFEU=FEU\times Y_{t}$$

Where *Y_t_* is the biomass of the *t^th^* harvest.

Coupling refers to the process of mutual interaction and influence between two or more factors, reflecting the system’s evolution from disorder to order (Shi et al., 2020). In this study, the Coupling Coordination Degree Model was employed to analyze the interaction between forage productivity and nutritive value. The calculation formulas are as follows:


$$\mu =\sum_{i=1}^{n}X_{i}\times W_{i}$$

$$u=\sum_{i=1}^{n}X_{i}\times W_{i}$$

$$C=2\sqrt{\frac{\mu \times u}{\mu +u}}$$

T=a×μ+b×u

$$D=\sqrt{C\times T}$$

Where *C* is the coupling degree, and a larger *C* value indicates a higher degree of coupling; *T* represents the coordination index; *a* and *b* are undetermined coefficients. Given that forage productivity and nutritive value are considered equally important, both *a* and *b* were set to 0.5 (*a*=*b*=0.5). *μ* is the forage productivity index, which is derived from the standardized dry matter yield of each harvest. *u* represents the forage nutritive value index, calculated through the weighted summation of the standardized values of WSC, DMD, EE, Ash, CP, and RFV. *D* denotes the coupling coordination degree, where a larger *D* value signifies a higher comprehensive quality of the forage (**Table 3**).

**Table 3:** Classification standards for the coupling coordination degree (D).

Coupling coordination degree (D)	Level of coupling coordination
0 ≤ D ≤ 0.1	Extreme incoordination
0.1< D ≤ 0.2	Severe incoordination
0.2< D ≤ 0.3	Moderate incoordination
0.3< D ≤ 0.4	Mild incoordination
0.4< D ≤ 0.5	On the verge of incoordination
0.5< D ≤ 0.6	Barely coordinated
0.6< D ≤ 0.7	Primary coordination
0.7< D ≤ 0.8	Intermediate coordination
0.8< D ≤ 0.9	Good coordination
0.9< D ≤ 1.0	Excellent coordination

The weight of each indicator (*W_i_*) was calculated using Principal Component Analysis (PCA) based on the variance contribution rates and the factor loading matrix (**Table 4**). The formula is as follows:

**Table 4:** Weights of nutritional indicators derived from principal component analysis.

Nutritional indicator	Weight (%)
WSC	18.61
DMD	11.99
EE	17.71
Ash	17.52
CP	15.85
RFV	18.32


$$z_{i}=\sum_{j=1}^{m}\left[W(PC_{j})\times |l_{ij}|\right]$$

$$W_{i}=z_{i}/\sum_{i=1}^{n}z_{i}$$

Where *W_i_* is the final weight of the *i^th^* indicator, *i* is the indicator index, and *n* is the total number of indicators; *W(PC_j_)* is the variance contribution rate of the *j^th^* principal component; *l_ij_* is the factor loading of the *i^th^* indicator on the *j^th^* principal component, *j* is the principal component index, and *m* is the total number of principal components; and *z_i_* is the comprehensive coefficient of the *i^th^* indicator.

## 3 Results

### 3.1 Productivity and stability

#### 3.1.1 Temporal dynamics of dry matter yield

**Figure 2:**
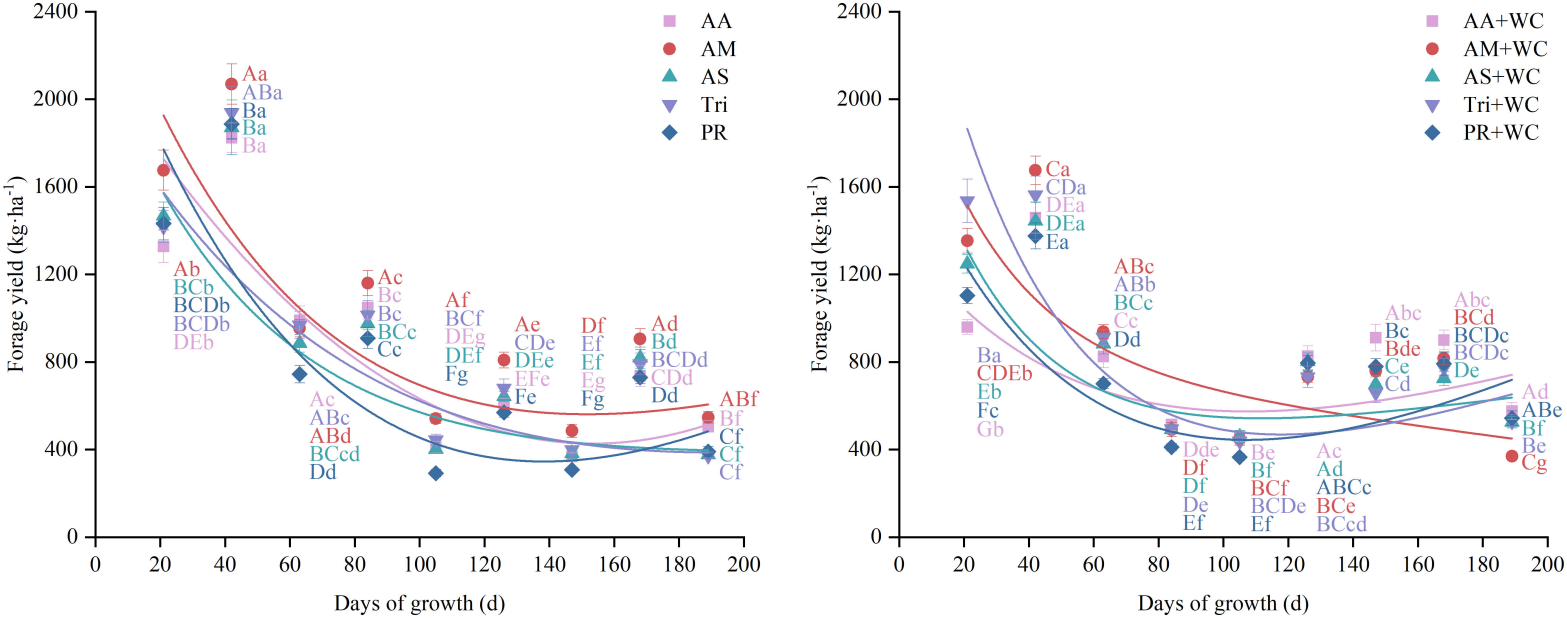
Growth curves and yield variation of high-sugar perennial ryegrass cultivars and their mixtures with white clover. Letters are color-coded to match the respective treatments. Different uppercase letters indicate significant differences among treatments at the same harvest time (*P*<0.05); different lowercase letters indicate significant differences across harvest times for the same treatment (*P*<0.05). The same notation applies to similar scatter plots with fitted curves.

As detailed in **Table 5** and **Figure 2**, the temporal dynamics of DM yield across all treatments were well-described by an exponential-linear regression model (*R*^2^ = 0.397–0.699). The growth trajectories were characterized by a sharp early peak at the second harvest (42d) followed by a fluctuating downward trend. During the early- to mid-season (21–84 d), the pure grass treatments displayed a strong growth advantage. Specifically, AM recorded the highest yield of 2068.98 kg·ha^-1^ at 42d, significantly outperforming all other treatments except Tri (*P*<0.05). In contrast, a shift in dominance occurred during the mid- to late-season (e.g., 126–147 d), where the grass-legume mixtures exhibited superior productivity and effectively buffered the late-season decline. At 147d, for instance, the grass-legume mixtures maintained robust yields ranging from 658.89 to 910.83 kg·ha^-1^, whereas pure grass treatments experienced a sharp drop to 308.23–484.97 kg·ha^-1^. The grass-legume mixtures produced significantly higher biomass than the pure grass treatments during this period (*P*<0.05).

**Table 5:** Fitting parameters of the regression curves for forage yield.

Treatment	p_1_	p_2_	p_3_	p_4_	R^2^
AA	114396	822.5	-112209	115.41	0.687
AM	3283	69.1	-607	5.29	0.617
AS	1998	49.6	251	0.54	0.646
Tri	2671	90.2	-617	3.57	0.699
PR	4843	90.6	-2313	11.62	0.572
AA+WC	1630	64.7	-249	4.77	0.397
AM+WC	1551	28.7	808	-1.90	0.528
AS+WC	1906	33.4	252	2.01	0.607
Tri+WC	3439	42.1	-327	4.98	0.641
PR+WC	2638	60.0	-788	7.37	0.573

The regression model is expressed as the following equation: $y=p_{1}e^{\left(-x/p_{2}\right)}+p_{3}+p_{4}x$.

#### 3.1.2 Total dry matter yield

**Figure 3:**
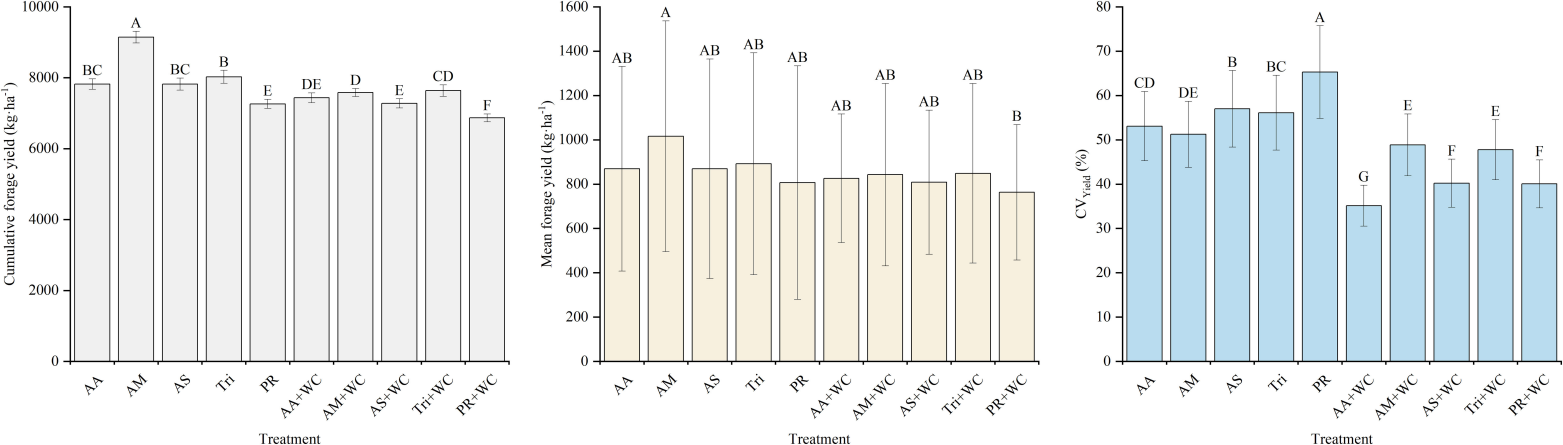
Cumulative forage yield, mean forage yield and yield stability of high-sugar perennial ryegrass cultivars and their mixtures with white clover. Different uppercase letters indicate significant differences among treatments (*P*<0.05). The same notation applies to similar bar charts.

Both grass component and sowing mode significantly influenced total dry matter yield (**Figure 3**). Among all treatments, AM produced the highest biomass, reaching 9144.48 kg·ha^-1^, which was significantly higher than that of all other treatments (*P*<0.05). This was followed by Tri, with a yield of 8025.40 kg·ha^-1^. In contrast, PR and PR+WC recorded the lowest yields among the pure grass and mixture treatments (7262.51 and 6867.54 kg·ha^-1^), with PR+WC yielding significantly less than PR (*P*<0.05).

#### 3.1.3 Yield stability

The grass-legume mixtures significantly outperformed the pure grass treatments in terms of yield stability (**Figure 3**). Specifically, the CV values for AA+WC (35.14%), AS+WC (40.20%), and PR+WC (40.06%) were significantly lower than those of their respective pure grass counterparts (*P*<0.05). Among all treatments, AA+WC proved to be the most stable, while PR was the least stable, recording the highest CV of 65.28%.

### 3.2 Community composition and competition

#### 3.2.1 Temporal dynamics and succession of community components

**Figure 4:**
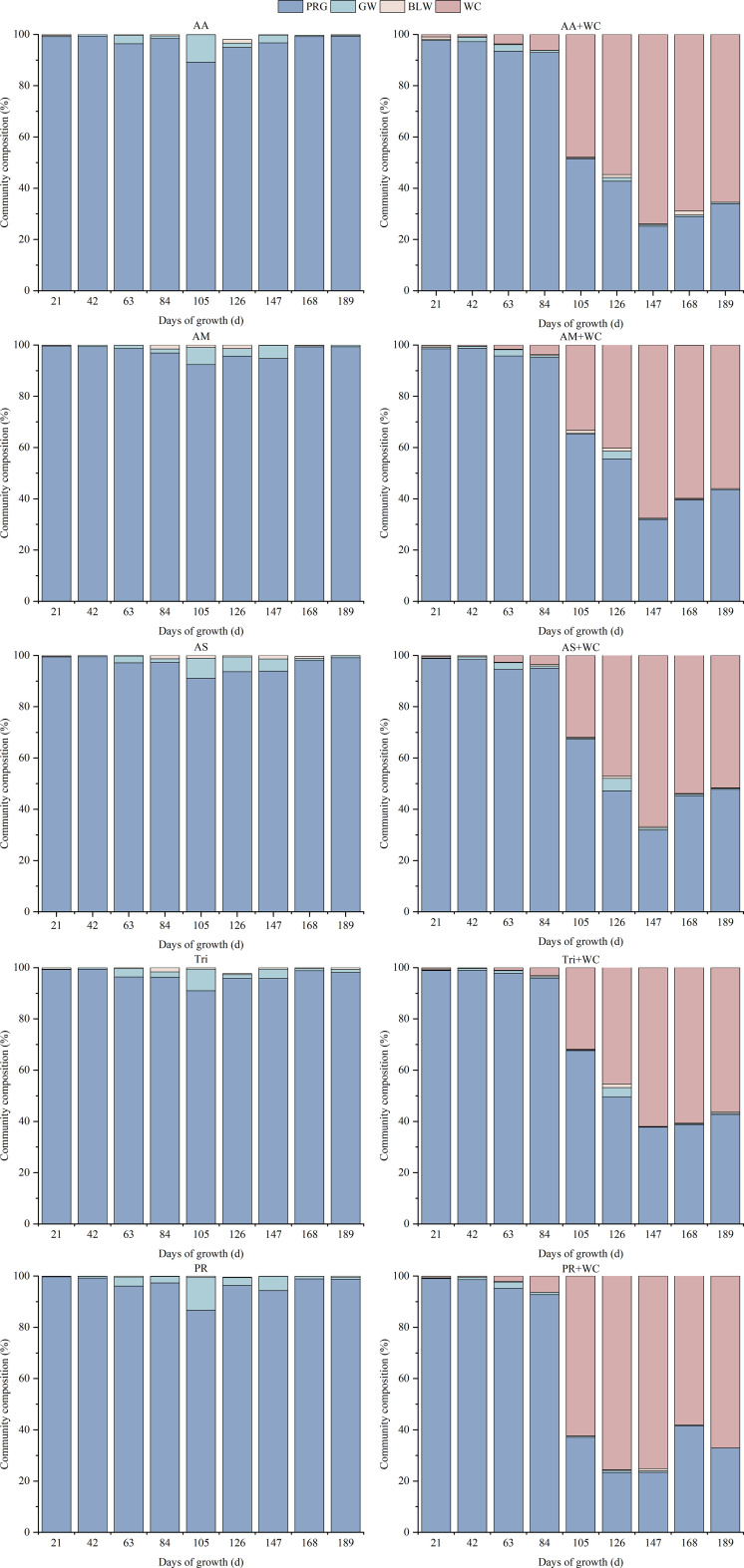
Community composition dynamics of high-sugar perennial ryegrass cultivars and their mixtures with white clover.

Sowing mode markedly altered the seasonal successional trajectory of the grassland community (**Figure 4**). In the pure grass stands, PRG maintained absolute dominance throughout the growing season, with its proportion ranging from 86% to 99%, although minor weed invasion was observed at 105d. Conversely, the grass-legume mixtures exhibited a complementary bimodal pattern. During 21–84 d, PRG remained the dominant species (proportion > 90%), while WC occupied a subordinate position (< 7%). However, a drastic reversal in community structure occurred at 105d. Driven by rising temperatures, WC grew rapidly, causing its proportion to surge to 30%–62%. By 126–189 d, WC replaced PRG as the dominant or co-dominant species. For instance, in the PR+WC, the WC proportion peaked at approximately 75% between 126 and 147d, while PRG declined to around 23%. Subsequently, the proportion of PRG recovered slightly during 168–189 d as temperatures decreased.

#### 3.2.2 Weed invasion and biological suppression

The grass-legume mixtures demonstrated substantial biological weed suppression capabilities (**Figure 4**). At 105d, the proportion of GW in PR reached 12.91%, followed by AA (10.77%) and AS (7.73%). In contrast, the GW proportion in all corresponding grass-legume mixtures remained below 0.5% during the same period.

#### 3.2.3 Stability of ryegrass populations

**Figure 5:**
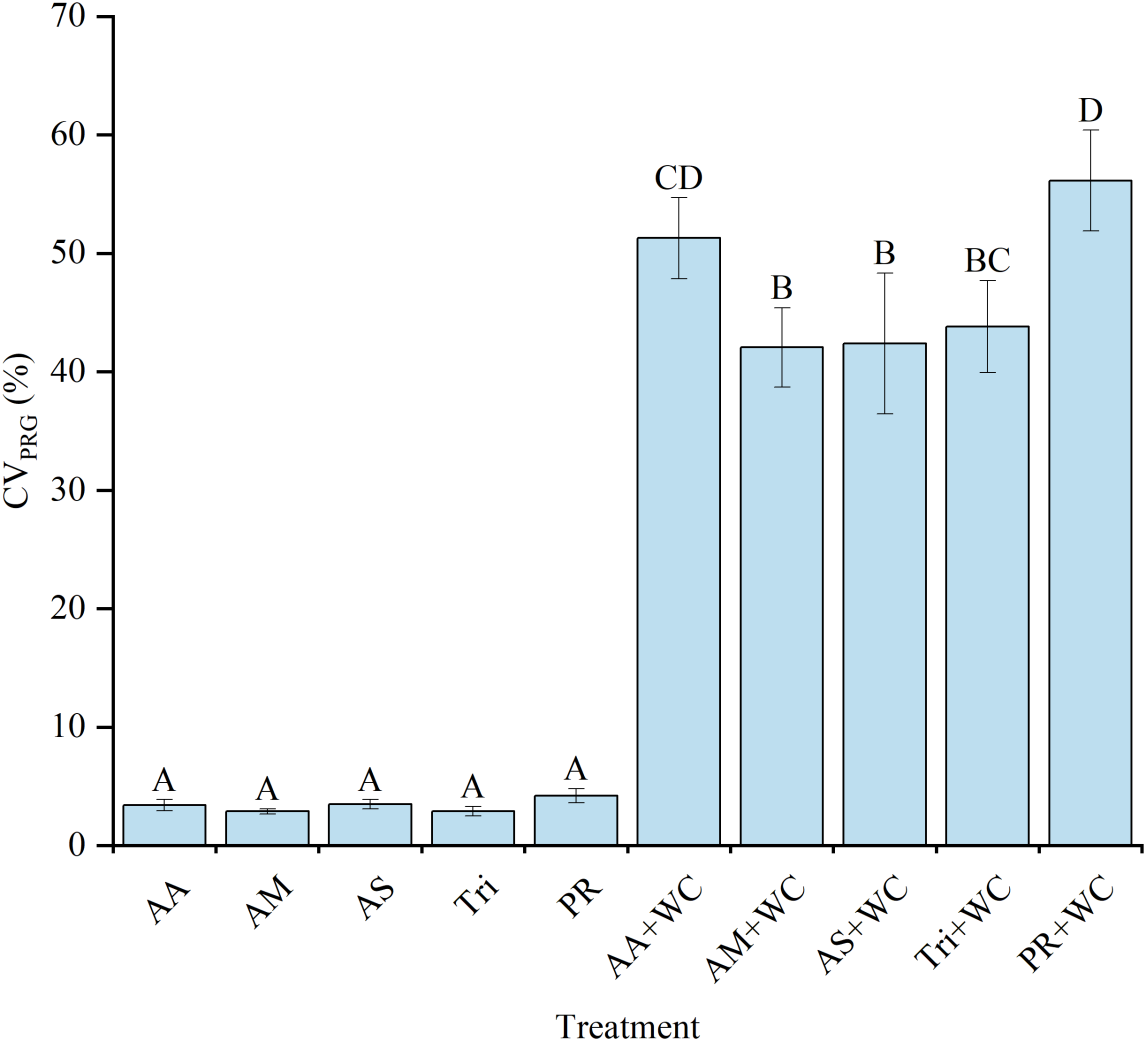
CV of the perennial ryegrass component of high-sugar perennial ryegrass cultivars and their mixtures with white clover.

Different grass cultivars exhibited varying competitive dynamics when mixed with WC (**Figure 5**). The CV for PRG proportion was remarkably low across all pure grass treatments (2.89%–4.21%) but increased substantially in the grass-legume mixtures (42.06%–56.14%) (*P*<0.05). Within the grass-legume mixtures, the HSG cultivar AberMagic demonstrated superior stability compared to the standard variety Premium. Specifically, PR+WC recorded the highest CV for PRG proportion (56.14%), whereas AM+WC exhibited a significantly lower CV (42.06%) (*P*<0.05).

### 3.3 Nutritive value and stability

**Figure 6:**
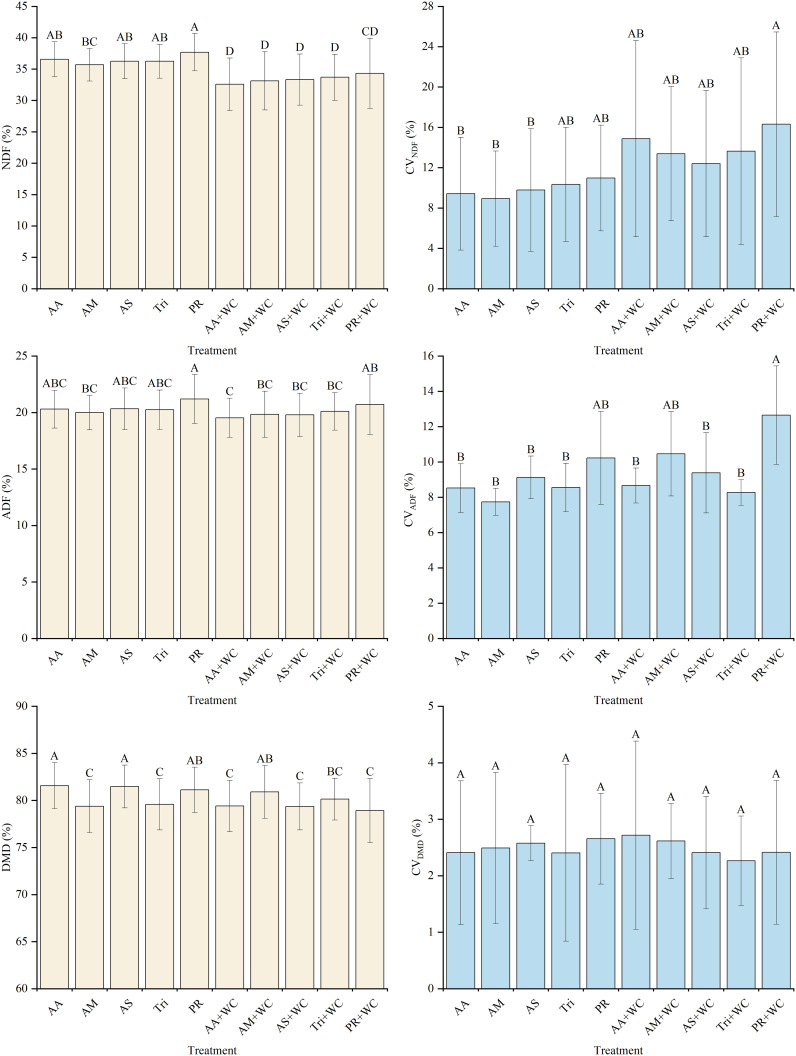
Mean values and stability of NDF, ADF, and DMD of high-sugar perennial ryegrass cultivars and their mixtures with white clover.

**Figure 7:**
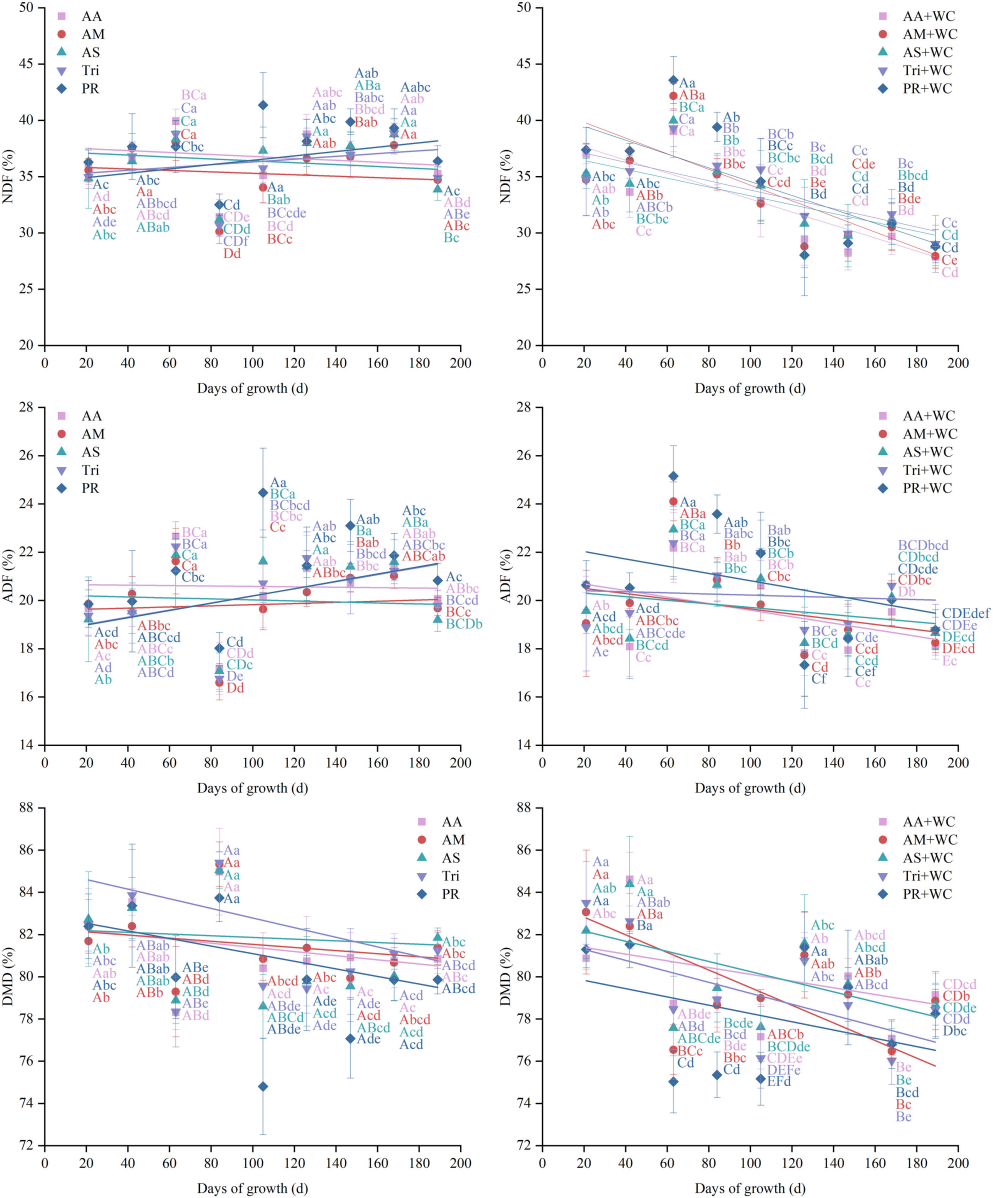
Temporal changes in NDF, ADF, and DMD of high-sugar perennial ryegrass cultivars and their mixtures with white clover.

Across the nine harvests, the grass-legume mixtures exhibited significantly lower overall mean NDF content (32.58%–34.33%) compared to the pure grass treatments (35.68%–37.69%) (*P*<0.05) (**Figure 6**). Linear regression analysis revealed distinct temporal NDF dynamics driving this difference (**Table 6**; **Figure 7**). The pure grass treatments exhibited no clear linear trend (*R*^2^ = 0.037–0.154), whereas the grass-legume mixtures demonstrated a pronounced linear decrease over time (R^2^ = 0.629–0.874). During the early-to-mid season (21–105 d), NDF content fluctuated, with the mixtures generally maintaining higher levels by 84d (35.18%–39.40%) compared to the pure grass treatments (30.11%–32.50%). Starting from 126d, NDF levels in the pure grass treatments remained consistently high (33.86%–39.32%), while the mixtures experienced a drastic decline, maintaining significantly lower concentrations of 27.85%–31.66% through 189d. The coefficient of variation (CV) for the mixtures was notably higher (12.40%–16.31%) compared to the pure grass treatments (8.93%–10.98%), with the PR+WC treatment recording the highest CV (16.31%), significantly exceeding those of the pure grass AA, AM, and AS treatments (*P*<0.05).

**Table 6:** Fitting parameters of the regression curves for NDF.

Treatment	A	B	R^2^
AA	37.66	-0.0087	0.06
AM	35.94	-0.0064	0.049
AS	37.25	-0.0084	0.037
Tri	35.07	0.0124	0.064
PR	34.51	0.0194	0.154
AA+WC	38.79	-0.0578	0.874
AM+WC	41.21	-0.0696	0.683
AS+WC	37.22	-0.0393	0.677
Tri+WC	37.92	-0.0412	0.629
PR+WC	40.71	-0.0615	0.669

The regression model is expressed as the following equation: y=A+Bx.

The grass-legume mixtures exhibited lower overall mean ADF content (19.53%–20.71%) compared to the pure grass treatments (19.99%–21.19%) (*P*<0.05) (**Figure 6**). The pure grass treatments exhibited no clear linear trend (*R*^2^ = 0.002–0.233), whereas the grass-legume mixtures demonstrated a weak-to-moderate linear decrease over time (*R*^2^ = 0.013–0.557) (**Table 7**; **Figure 7**). During the early-to-mid season (21–105 d), ADF content fluctuated, with the mixtures generally maintaining higher levels by 84d (20.65%–23.57%) compared to the sharp decline in the pure grass treatments (16.59%–18.02%). However, starting from 126d, ADF levels in the pure grass treatments rebounded and remained consistently high (19.20%–23.09%), while the mixtures experienced a decline, maintaining lower concentrations of 17.33%–20.61% through 189d. The coefficient of variation (CV) for the mixtures was generally higher (8.27%–12.65%) compared to the pure grass treatments (7.74%–10.23%), with the PR+WC treatment recording the highest CV (12.65%), significantly exceeding those of most pure grass treatments (*P*<0.05).

**Table 7:** Fitting parameters of the regression curves for ADF.

Treatment	A	B	R^2^
AA	20.67	-0.0009	0.002
AM	19.59	0.0024	0.018
AS	20.23	-0.0021	0.006
Tri	18.72	0.0147	0.148
PR	18.68	0.0151	0.233
AA+WC	20.96	-0.0136	0.557
AM+WC	20.72	-0.0107	0.272
AS+WC	20.46	-0.0075	0.176
Tri+WC	20.46	-0.0023	0.013
PR+WC	22.33	-0.0151	0.198

The regression model is expressed as the following equation: y=A+Bx.

The pure grass treatments generally exhibited slightly higher overall mean DMD content (79.38%–81.57%) compared to the grass-legume mixtures (78.92%–80.90%) (*P*<0.05) (**Figure 6**). Linear regression analysis indicated an overall decreasing trend in DMD for all treatments, though with varying intensities (**Table 8**; **Figure 7**). The pure grass treatments exhibited weak linear fits (*R*^2^ = 0.014–0.261), whereas the grass-legume mixtures demonstrated slightly stronger negative linear trends over time (*R*^2^ = 0.186–0.825). During the early-to-mid season (21–105 d), DMD content fluctuated, with the pure grass treatments reaching a distinct peak by 84d (83.73%–85.40%), which was significantly higher than the mixtures (75.34%–79.46%). Starting from 126d, DMD levels in the pure grass treatments remained relatively high (79.84%–81.84% across 168–189 d), while the mixtures experienced a further decline to lower concentrations of 76.02%–79.14%. Unlike fiber content, the temporal stability of DMD was highly consistent across all treatments; the CV remained universally low (2.26%–2.71%), with no significant differences observed between the mixtures and the pure grass treatments (*P* > 0.05).

**Table 8:** Fitting parameters of the regression curves for DMD.

Treatment	A	B	R^2^
AA	82.38	-0.0099	0.107
AM	82.27	-0.0074	0.138
AS	82.27	-0.0040	0.014
Tri	85.06	-0.0228	0.206
PR	82.89	-0.0180	0.261
AA+WC	81.71	-0.0160	0.289
AM+WC	83.66	-0.0418	0.825
AS+WC	82.64	-0.0240	0.385
Tri+WC	81.82	-0.0260	0.362
PR+WC	80.22	-0.0196	0.186

The regression model is expressed as the following equation: y=A+Bx.

**Figure 8:**
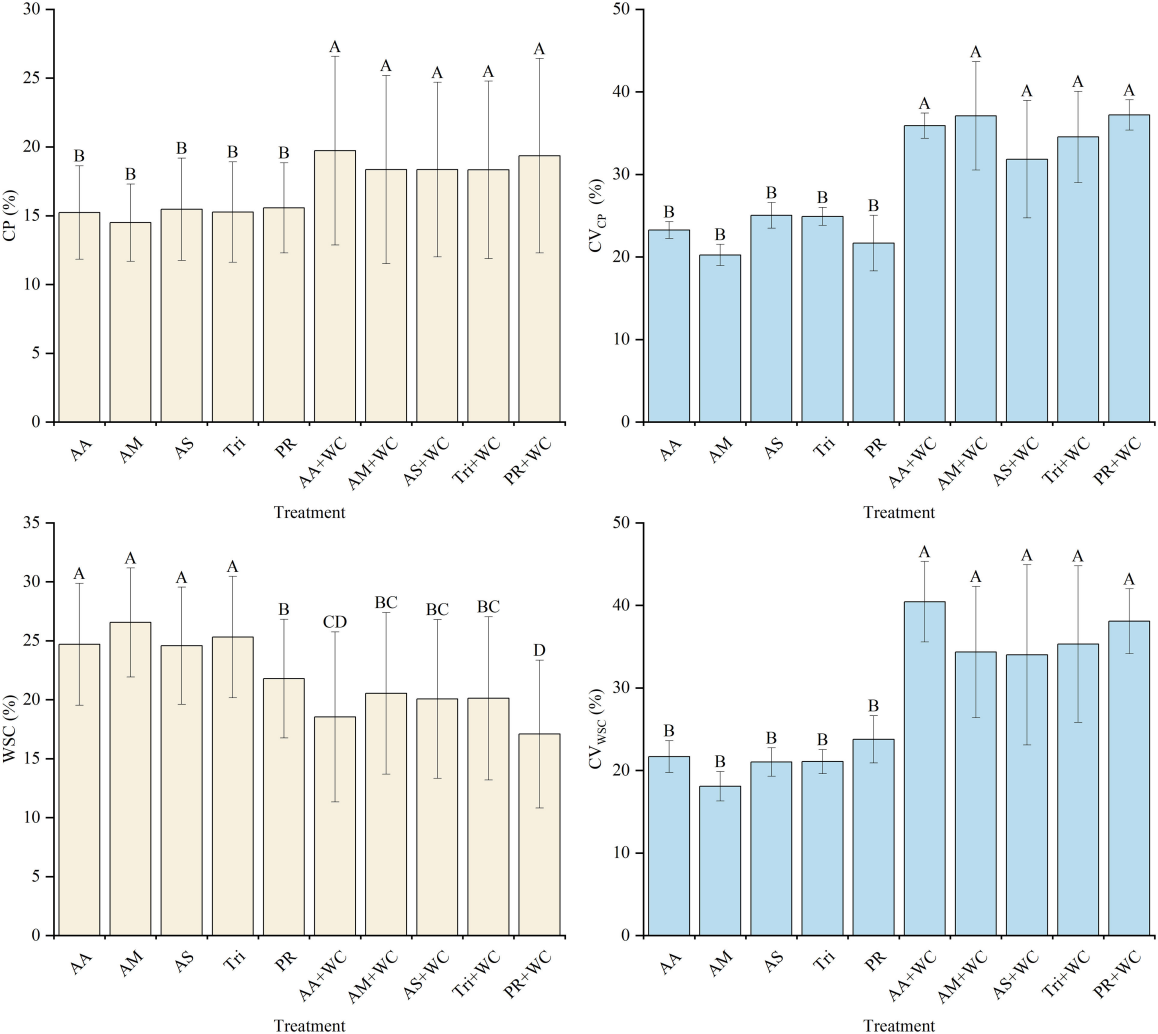
Mean values and stability of CP and WSC of high-sugar perennial ryegrass cultivars and their mixtures with white clover.

**Figure 9:**
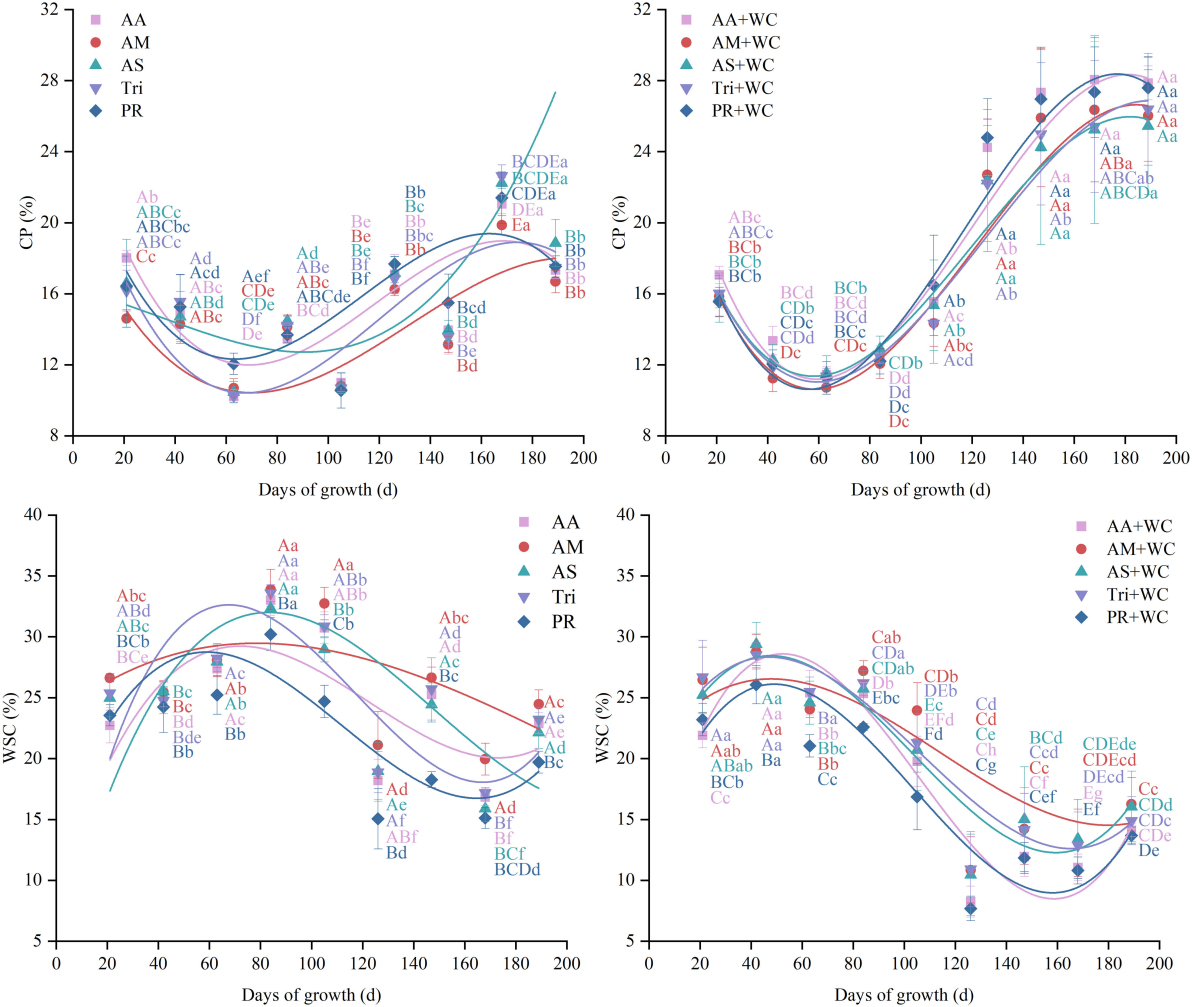
Temporal changes in CP and WSC of high-sugar perennial ryegrass cultivars and their mixtures with white clover.

The grass-legume mixtures exhibited significantly higher overall mean CP content (18.33%–19.72%) compared to the pure grass treatments (14.49%–15.57%) (*P*<0.05) (**Figure 8**). Cubic polynomial regression analysis revealed that the temporal dynamics of all treatments followed a non-linear “fall-rise-fall” trajectory (**Table 9**; **Figure 9**). The pure grass treatments exhibited moderate model fits (*R*^2^ = 0.582–0.763), whereas the grass-legume mixtures demonstrated exceptionally good model fits (*R*^2^ = 0.968–0.979). During the early season (21–84 d), CP content in the pure grass treatments was generally slightly higher than in the mixtures, reaching 13.46%–14.47% by 84d compared to the mixtures (12.06%–12.89%). Starting from 105d, CP levels in the pure grass treatments remained relatively lower (fluctuating between 10.55% and 22.63% across 105–189 d), while the mixtures experienced a drastic late-season surge, maintaining significantly higher concentrations of 22.20%–28.05% through 126–189 d. The CV for the mixtures was significantly higher (31.84%–37.21%) compared to the pure grass treatments (20.24%–25.03%) (*P*<0.05).

**Table 9:** Fitting parameters of the regression curves for CP.

Treatment	A	B_1_	B_2_ (×10^-3^)	B3 (×10^-5^)	R^2^
AA	26.71	-0.4938	5.04	-1.41	0.744
AM	20.65	-0.3322	3.23	-0.82	0.582
AS	16.23	-0.0313	-0.60	0.57	0.757
Tri	24.87	-0.4924	5.07	-1.40	0.66
PR	24.55	-0.4434	4.86	-1.43	0.763
AA+WC	27.23	-0.6085	6.82	-1.89	0.976
AM+WC	24.32	-0.5224	5.87	-1.61	0.974
AS+WC	23.88	-0.4839	5.51	-1.53	0.979
Tri+WC	24.00	-0.4876	5.39	-1.45	0.976
PR+WC	25.55	-0.5965	7.02	-2.01	0.968

The regression model is expressed as the following equation: $y=A+B_{1}\cdot x+B_{2}\cdot x^{2}+B_{3}\cdot x^{3}$.

The pure grass treatments exhibited significantly higher overall mean WSC content (21.79%–26.55%) compared to the grass-legume mixtures (17.09%–20.54%) (*P*<0.05) (**Figure 8**). Cubic polynomial regression analysis revealed an overall “rise-fall-rise” trajectory, opposite to the trend of CP (**Table 10**; **Figure 9**). The pure grass treatments exhibited moderate to strong model fits (*R*^2^ = 0.384–0.832), whereas the grass-legume mixtures generally demonstrated stronger non-linear temporal trends (*R*^2^ = 0.597–0.928). During the early season (21–84 d), WSC content in all treatments increased, with the pure grass treatments reaching a distinct peak by 84d (30.22%–33.89%), which was significantly higher than the mixtures (22.59%–27.20%). Starting from 105d, a significant divergence occurred as WSC levels dropped across all treatments: WSC levels in the pure grass treatments remained relatively higher (fluctuating between 15.05% and 32.73% across 105–189 d), while the mixtures experienced a drastic late-season decline, maintaining significantly lower concentrations of 7.69%–23.95% through 105–189 d. The CV for the mixtures was significantly higher (34.00%–40.44%) compared to the pure grass treatments (18.08%–23.77%) (*P*<0.05).

**Table 10:** Fitting parameters of the regression curves for WSC.

Treatment	A	B_1_	B_2_	B_3_ (×10^-5^)	R^2^
AA	8.64	0.665	-0.0065	1.78	0.619
AM	23.50	0.165	-0.0013	0.21	0.384
AS	3.62	0.785	-0.0066	1.48	0.810
Tri	3.25	1.005	-0.0105	2.97	0.831
PR	13.50	0.589	-0.0068	2.03	0.832
AA+WC	9.15	0.835	-0.0106	3.35	0.928
AM+WC	20.63	0.271	-0.0036	1.05	0.597
AS+WC	17.12	0.528	-0.0072	2.32	0.858
Tri+WC	18.97	0.441	-0.0060	1.89	0.902
PR+WC	12.63	0.614	-0.0082	2.64	0.874

The regression model is expressed as the following equation: $y=A+B_{1}\cdot x+B_{2}\cdot x^{2}+B_{3}\cdot x^{3}$.

**Figure 10:**
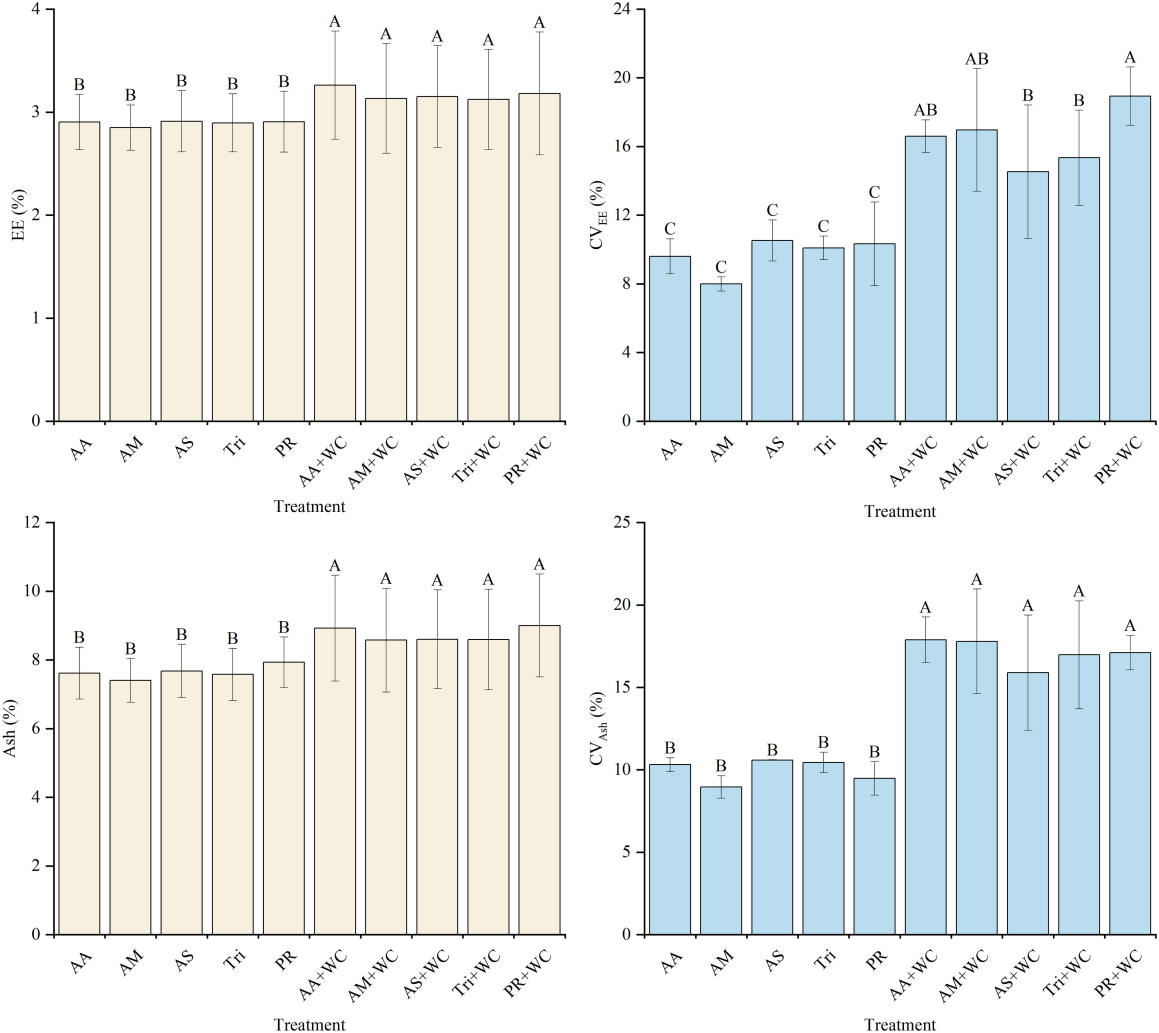
Mean values and stability of EE and Ash of high-sugar perennial ryegrass cultivars and their mixtures with white clover.

**Figure 11:**
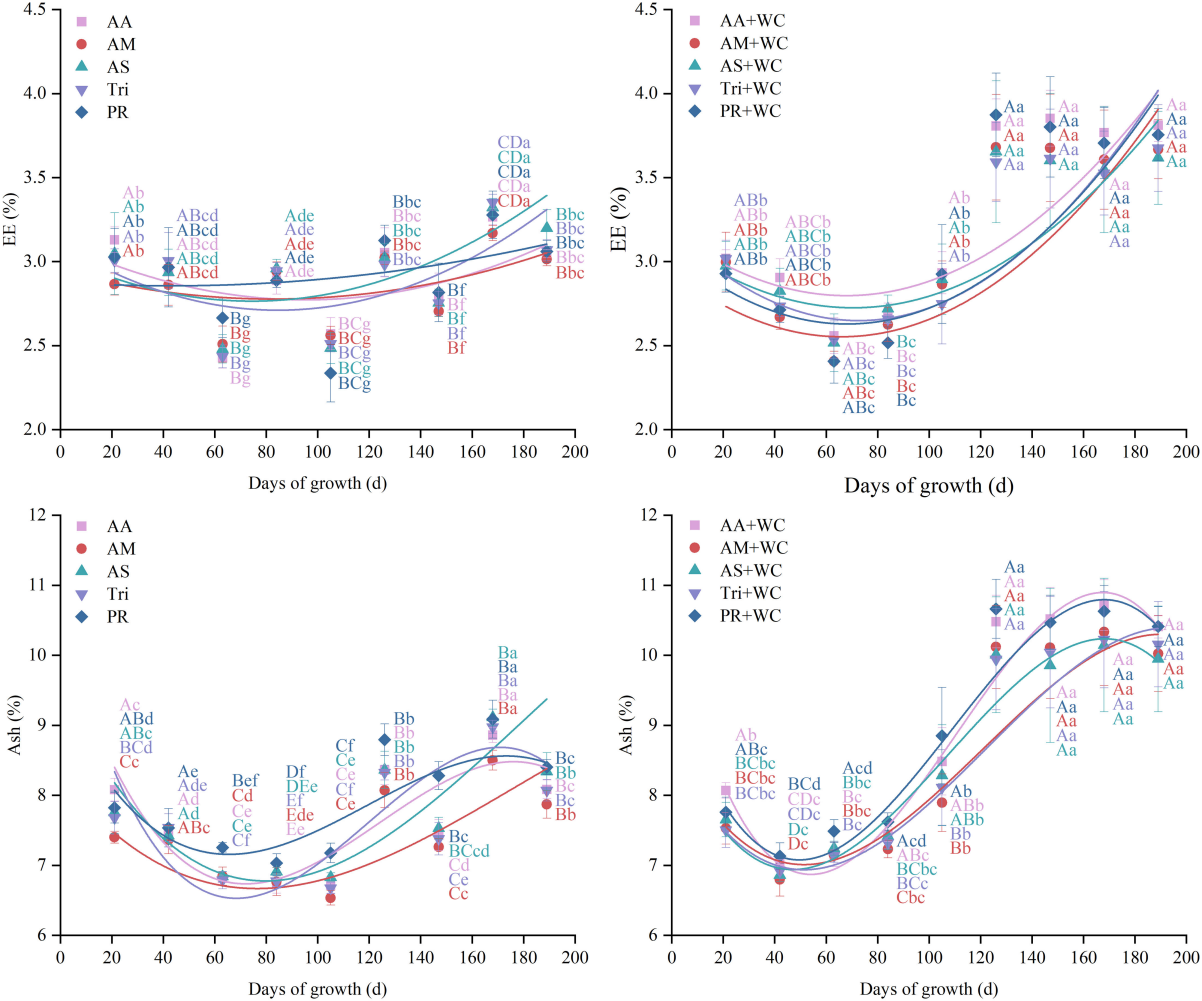
Temporal changes in EE and Ash of high-sugar perennial ryegrass cultivars and their mixtures with white clover.

The grass-legume mixtures exhibited significantly higher overall mean EE content (3.12%–3.26%) compared to the pure grass treatments (2.85%–2.91%) (*P*<0.05) (**Figure 10**). Quadratic polynomial regression analysis revealed an overall “slow fall-large rise” trajectory (**Table 11**; **Figure 11**). The pure grass treatments exhibited weak to moderate model fits (*R*^2^ = 0.213–0.480), whereas the grass-legume mixtures demonstrated consistently good model fits (*R*^2^ = 0.743–0.784). During the early-to-mid season (21–105 d), the pure grass treatments generally maintained slightly higher levels by 84d (2.88%–2.95%) compared to the mixtures (2.51%–2.72%). Starting from 126d, EE levels in the pure grass treatments remained relatively lower (fluctuating between 2.70% and 3.35% across 126–189 d), while the mixtures experienced a drastic late-season surge, maintaining significantly higher concentrations of 3.52%–3.87% through 126–189 d. The CV for the mixtures was significantly higher (14.52%–18.93%) compared to the pure grass treatments (7.99%–10.52%) (*P*<0.05).

**Table 11:** Fitting parameters of the regression curves for EE.

Treatment	A	B_1_	B_2_ (×10^-5^)	R^2^
AA	3.11	-0.0072	3.75	0.331
AM	2.94	-0.0040	2.43	0.213
AS	3.03	-0.0071	4.79	0.480
Tri	3.10	-0.0093	5.48	0.439
PR	2.88	-0.0010	1.17	0.302
AA+WC	3.18	-0.0112	8.26	0.748
AM+WC	2.94	-0.0118	8.93	0.746
AS+WC	3.12	-0.0112	7.93	0.752
Tri+WC	3.18	-0.0147	10.12	0.784
PR+WC	3.06	-0.0127	9.32	0.743

The regression model is expressed as the following equation: $y=A+B_{1}x+B_{2}x^{2}$.

The grass-legume mixtures exhibited significantly higher overall mean Ash content (8.58%–9.00%) compared to the pure grass treatments (7.40%–7.93%) (*P*<0.05) (**Figure 10**). Cubic polynomial regression analysis revealed an overall “fall-rise-fall” trajectory (**Table 12**; **Figure 11**). The pure grass treatments exhibited moderate to good model fits (*R*^2^ = 0.664–0.854), whereas the grass-legume mixtures demonstrated exceptionally good model fits (*R*^2^ = 0.869–0.987). During the early season (21–84 d), the pure grass treatments dropped to lower levels by 84d (6.73%–7.03%) compared to the mixtures (7.23%–7.62%). Starting from 126d, Ash levels in the pure grass treatments remained relatively lower (fluctuating between 7.26% and 9.11% across 126–189 d), while the mixtures experienced a drastic late-season surge, maintaining significantly higher concentrations of 9.85%–10.71% through 126–189 d. The CV for the mixtures was significantly higher (15.89%–17.88%) compared to the pure grass treatments (8.96%–10.58%) (*P*<0.05).

**Table 12:** Fitting parameters of the regression curves for Ash.

Treatment	A	B_1_	B_2_ (×10^-3^)	B_3_ (×10^-6^)	R^2^
AA	10.40	-0.1177	1.15	-3.10	0.854
AM	8.26	-0.0458	0.38	-0.72	0.664
AS	9.52	-0.0761	0.61	-1.14	0.839
Tri	10.69	-0.1405	1.44	-4.01	0.785
PR	9.35	-0.0765	0.81	-2.25	0.819
AA+WC	10.48	-0.1498	1.83	-5.52	0.987
AM+WC	8.7	-0.0721	0.89	-2.46	0.869
AS+WC	8.82	-0.0882	1.20	-3.71	0.894
Tri+WC	8.63	-0.0723	0.89	-2.43	0.921
PR+WC	9.57	-0.1116	1.46	-4.46	0.955

The regression model is expressed as the following equation: $y=A+B_{1}\cdot x+B_{2}\cdot x^{2}+B_{3}\cdot x^{3}$.

### 3.4 Comprehensive evaluation

#### 3.4.1 Temporal dynamics of RFV

**Figure 12:**
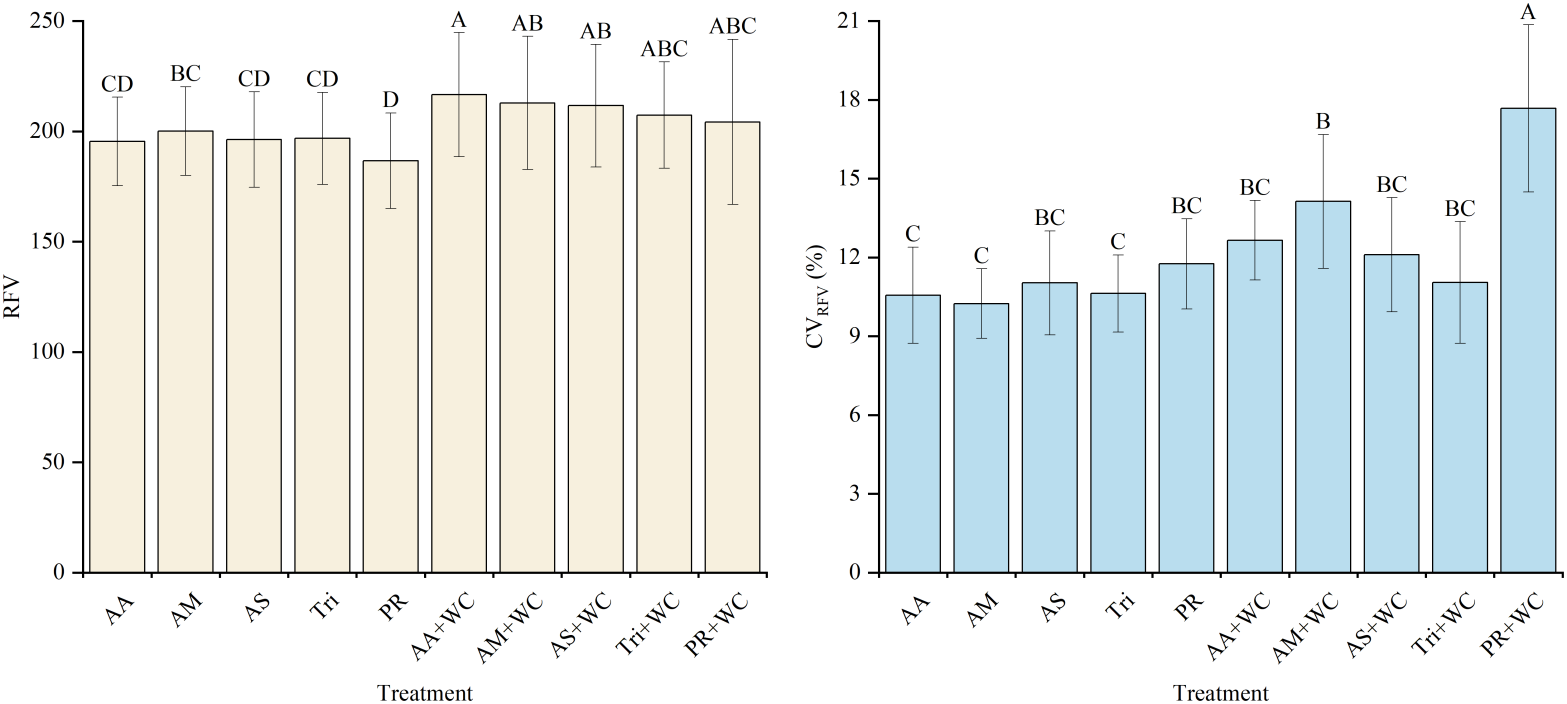
Mean values and stability of RFV of high-sugar perennial ryegrass cultivars and their mixtures with white clover.

**Figure 13:**
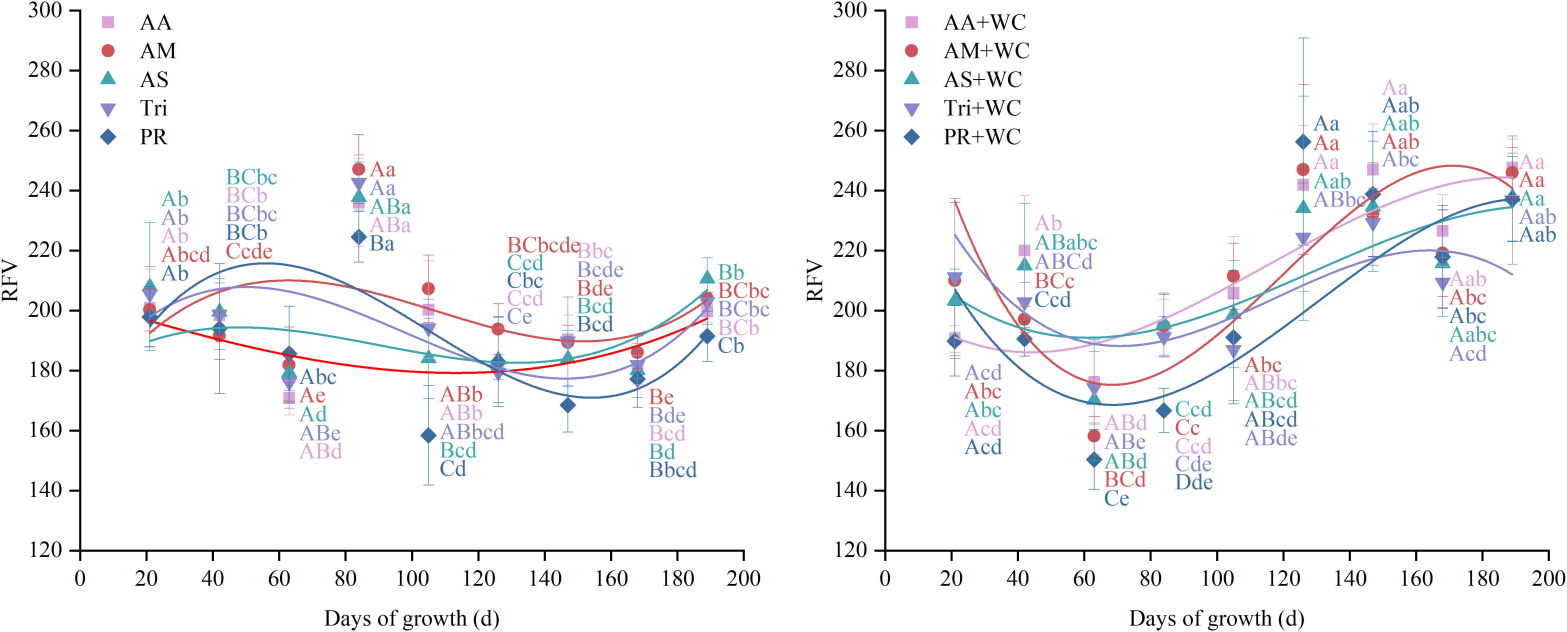
Temporal changes in RFV of high-sugar perennial ryegrass cultivars and their mixtures with white clover.

The grass-legume mixtures exhibited significantly higher overall mean RFV (204.27–216.62) compared to the pure grass treatments (186.71–200.13) (*P*<0.05) (**Figure 12**). Cubic polynomial regression analysis revealed differing temporal dynamics between treatments (**Table 13**; **Figure 13**). The pure grass treatments exhibited weak to moderate model fits (*R*^2^ = 0.1915–0.5350), whereas the grass-legume mixtures demonstrated better model fits (*R*^2^ = 0.6232–0.8454) and followed a distinct “fall-rise-fall” trajectory. During the early-to-mid season (21–105 d), RFV fluctuated, with the pure grass treatments reaching a distinct peak by 84d (224.49–247.05), significantly higher than the mixtures (166.68–195.44). Starting from 126d, RFV in the pure grass treatments remained relatively lower (fluctuating between 168.50 and 210.63 across 126–189 d), while the mixtures experienced a drastic late-season surge, maintaining significantly higher values of 209.33–256.24 through 126–189 d. The CV for the mixtures was significantly higher (11.05%–17.68%) compared to the pure grass treatments (10.24%–11.75%) (*P*<0.05).

**Table 13:** Fitting parameters of the regression curves for RFV.

Treatment	A	B_1_	B_2_ (×10^-3^)	B_3_ (×10^-5^)	R^2^
AA	203.52	-0.3428	0.37	0.67	0.2071
AM	165.52	1.6474	-18.58	5.78	0.3289
AS	178.08	0.7709	-10.93	4.05	0.1915
Tri	174.72	1.4975	-20.12	6.84	0.3693
PR	155.60	2.4464	-29.81	9.46	0.5350
AA+WC	206.07	-0.9860	13.84	-3.99	0.8454
AM+WC	315.97	-4.7480	48.59	-13.53	0.7673
AS+WC	225.84	-1.2742	13.67	-3.54	0.6979
Tri+WC	271.33	-2.7421	27.71	-7.86	0.6232
PR+WC	255.66	-2.8828	28.52	-7.30	0.7000

The regression model is expressed as the following equation: $y=A+B_{1}\cdot x+B_{2}\cdot x^{2}+B_{3}\cdot x^{3}$.

#### 3.4.2 Temporal dynamics of FEU and PFEU

**Figure 14:**
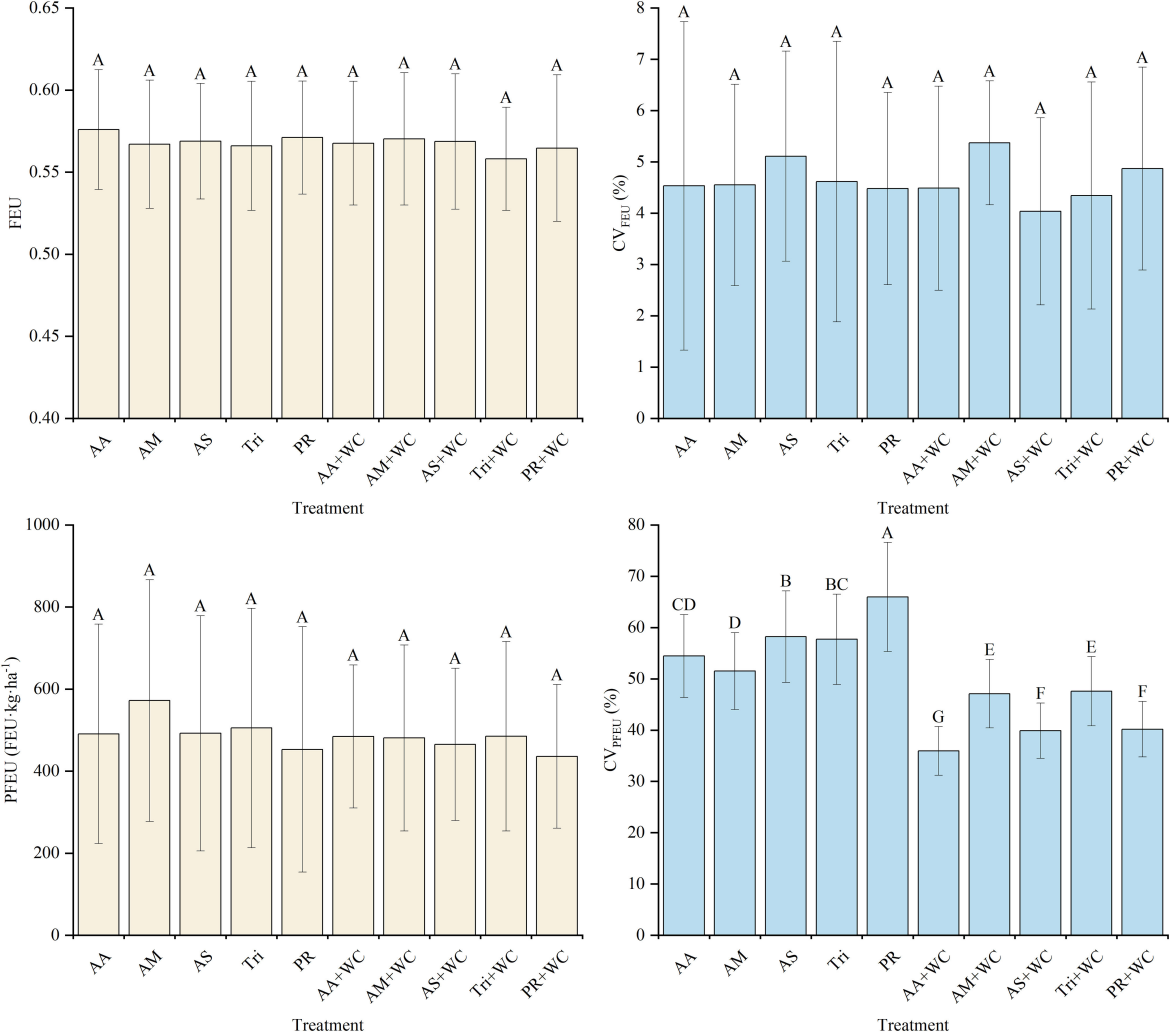
Mean values and stability of FEU and PFEU of high-sugar perennial ryegrass cultivars and their mixtures with white clover.

**Figure 15:**
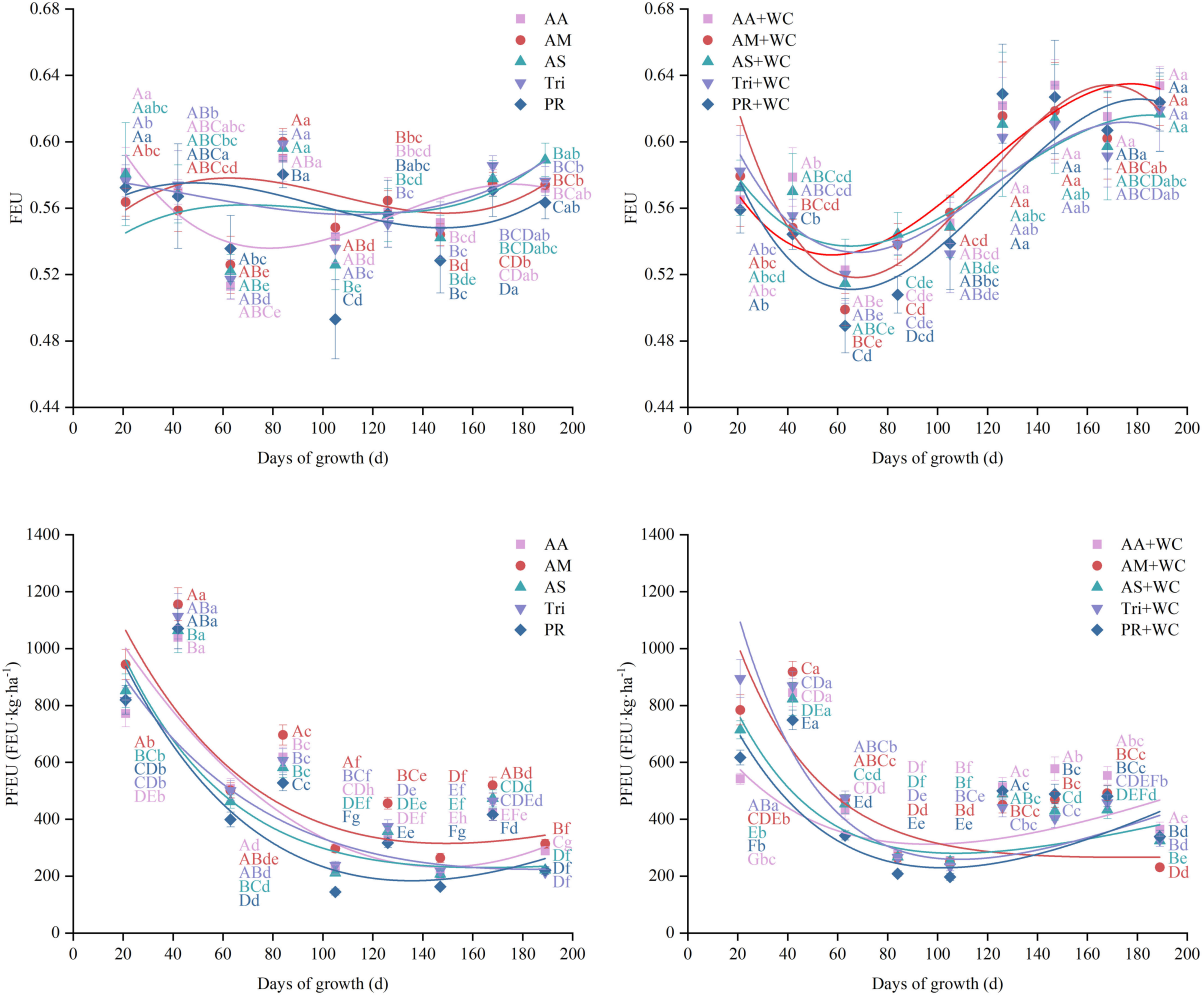
Temporal changes in FEU and PFEU of high-sugar perennial ryegrass cultivars and their mixtures with white clover.

There were no significant differences in the overall mean FEU and PFEU between the pure grass treatments and the grass-legume mixtures (*P* > 0.05) (**Figure 14**). Despite similar overall means, regression analyses revealed distinct temporal dynamics. For FEU, the mixtures demonstrated good cubic polynomial fits (*R*^2^ = 0.813–0.865) following a “fall-rise-fall” trajectory, whereas the pure grass treatments exhibited weak fits (*R*^2^ = 0.119–0.482) (**Table 14**; **Figure 15**). Conversely, both treatments exhibited moderate non-linear fits for PFEU (*R*^2^ = 0.433–0.690) with a “sharp fall-slight rebound” trajectory (**Table 15**; **Figure 15**). During the early season, the pure grass treatments reached higher peak values for both indices, maximizing PFEU at 42d (1039.42–1155.69) and FEU at 84d (0.58–0.60), which were significantly higher than the mixtures. Starting from 126d, a late-season reversal occurred: the pure grass treatments fluctuated at relatively lower levels, while the mixtures surged to maintain significantly higher FEU (0.59–0.63) and generally higher PFEU (230.79–577.55) through 189d. Regarding temporal stability, FEU was universally stable across all treatments with low CVs (4.03%–5.37%) and no significant differences (*P* > 0.05), whereas for PFEU, the mixtures exhibited a significantly lower CV (35.95%–47.57%) compared to the pure grass treatments (51.50%–65.96%) (*P*<0.05).

**Table 14:** Fitting parameters of the regression curves for FEU.

Treatment	A	B_1_	B_2_ (×10^-5^)	B_3_ (×10^-8^)	R^2^
AA	0.65	-0.0035	3.18	-8.33	0.482
AM	0.53	0.0018	-2.07	6.51	0.279
AS	0.52	0.0014	-1.58	5.41	0.153
Tri	0.58	-0.0002	-0.27	2.00	0.199
PR	0.55	0.0012	-1.61	5.49	0.119
AA+WC	0.63	-0.0037	4.22	-11.97	0.841
AM+WC	0.74	-0.0077	7.95	-22.4	0.865
AS+WC	0.63	-0.0033	3.44	-9.17	0.813
Tri+WC	0.67	-0.0046	4.68	-12.84	0.853
PR+WC	0.66	-0.0053	5.52	-14.95	0.813

The regression model is expressed as the following equation: $y=A+B_{1}\cdot x+B_{2}\cdot x^{2}+B_{3}\cdot x^{3}$.

**Table 15:** Fitting parameters of the regression curves for PFEU.

Treatment	p_1_	p_2_	p_3_	p_4_	R^2^
AA	23837	443.6	-22542	38.51	0.69
AM	1836	69.5	-358	3.08	0.563
AS	1410	47.4	36	0.92	0.541
Tri	1333	70.1	-125	1.38	0.646
PR	2347	80.3	-983	5.41	0.497
AA+WC	1023	59.1	-214	3.39	0.433
AM+WC	1431	33.8	218	0.23	0.539
AS+WC	1265	39.5	-31	2.12	0.667
Tri+WC	2165	38.2	-227	3.31	0.667
PR+WC	1603	56.7	-511	4.66	0.605

The regression model is expressed as the following equation: $y=p_{1}e^{\left(-x/p_{2}\right)}+p_{3}+p_{4}x$.

#### 3.4.3 Temporal dynamics of coupling coordination degree

**Figure 16:**
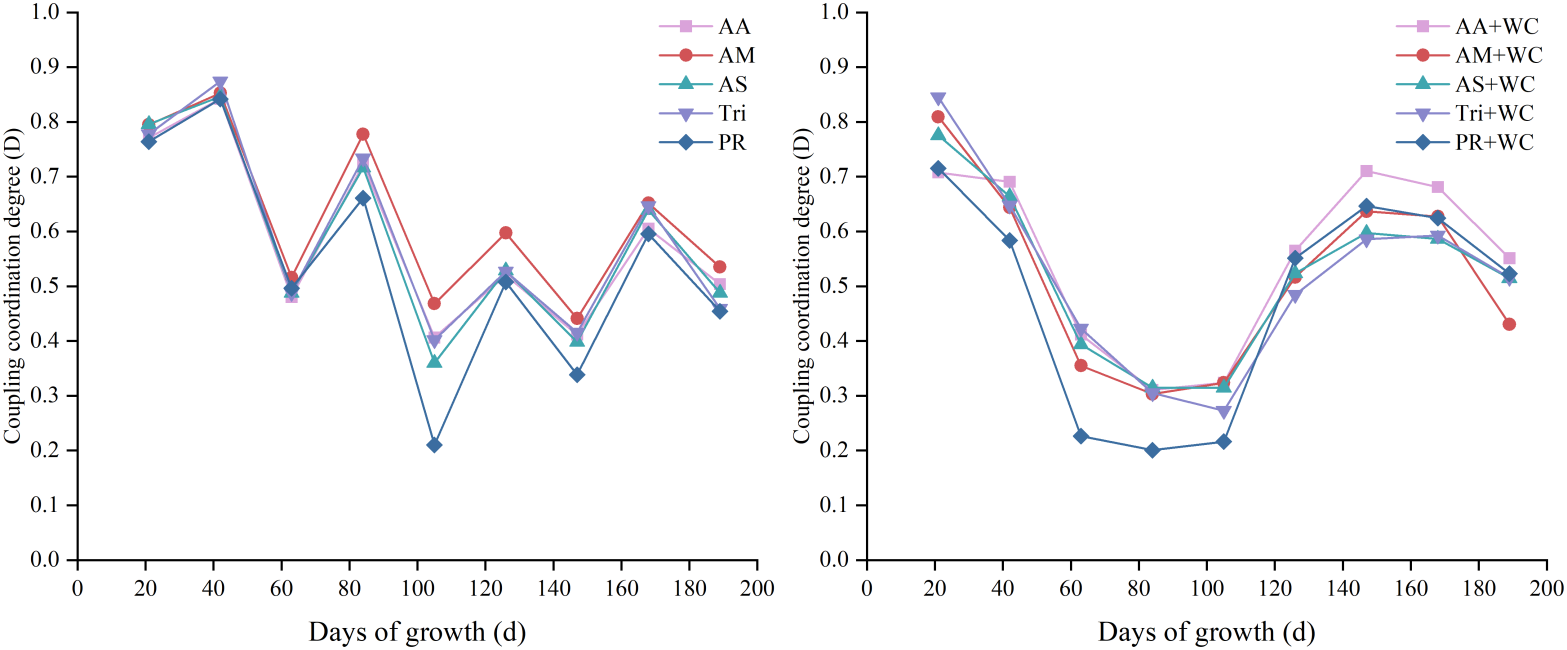
Temporal changes in coupling coordination degree (D) of high-sugar perennial ryegrass cultivars and their mixtures with white clover.

The pure grass treatments exhibited a wavy descending trend followed by a slow increase (**Figure 16**), whereas the grass-legume mixtures experienced a rapid decline in the early-to-mid season followed by a distinct late-season reversal. Comparing the two stands, the D values of the pure grass treatments were generally higher than those of the mixtures during the 42–105 d period; however, during the late season, particularly at 147d, the mixtures surpassed the pure grass treatments. Within the pure grass treatments, AM and Tri generally exhibited relatively better coupling coordination, while PR consistently showed the poorest coordination. Among the mixtures, AA+WC demonstrated relatively better coupling during the late-season rebound, whereas PR+WC showed the poorest coordination with the steepest declines. The pure grass treatments reached “good coordination” at 42d and a secondary peak of “primary to intermediate coordination” by 84d, before dropping to fluctuate between “moderate incoordination” and “primary coordination” across105–189 d. The grass-legume mixtures, after an initial “primary to good coordination” at 21d, dropped sharply to “severe to mild incoordination” by 84–105 d. Following the late-season reversal, the mixtures reached a peak of “barely coordinated” to “intermediate coordination” at 147–168 d.

### 3.5 Correlation analysis among indicators

**Figure 17:**
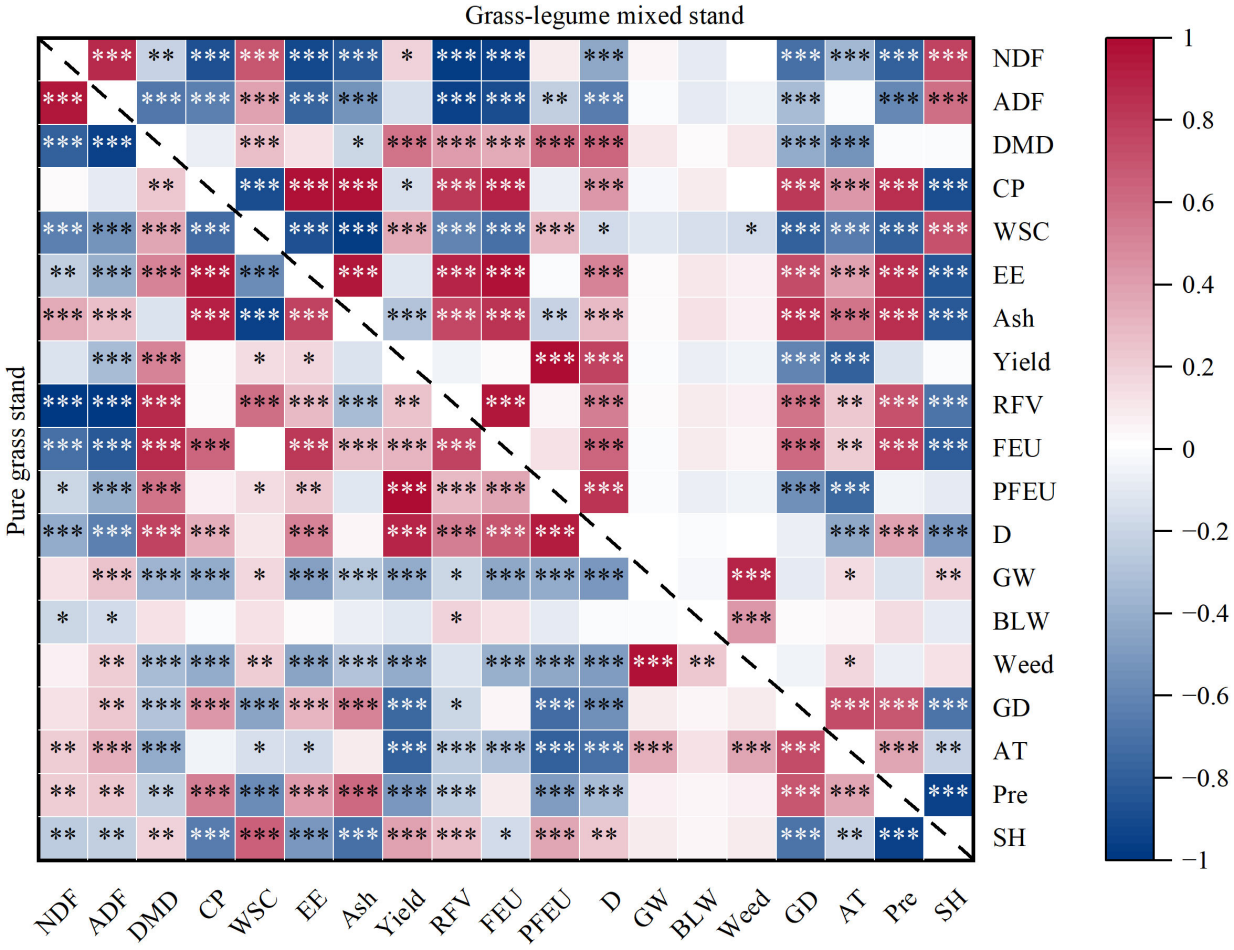
Correlation heatmaps among agronomic traits, weed proportions, and meteorological factors in monoculture grass (lower left) and grass-legume mixed (upper right) treatments. *P<0.05; **P<0.01; ***P<0.001. NDF, Neutral Detergent Fiber; ADF, Acid Detergent Fiber; DMD, Dry Matter Digestibility; CP, Crude Protein; WSC, Water Soluble Carbohydrates; EE, Ether Extract; Ash, Ash content; Yield, Forage Yield; RFV, Relative Feed Value; FEU, Forage Food Equivalent Unit; PFEU, Production of Forage Food Equivalent Unit; D, Coupling Coordination Degree; GW, Proportion of Grass Weeds; BLW, Proportion of Broad-leaved Weeds; Weed, Total Weed Proportion; GD, Growth Days; AT, Accumulated Temperature (≥0 C); Pre, Precipitation; SH, Sunshine Hours.

Pearson correlation analysis revealed fundamentally contrasting interrelationships between the pure grass treatments and the grass-legume mixtures (**Figure 17**). Regarding the relationship between productivity and quality, in pure grass stands, the Coupling Coordination Degree (D) was primarily driven by productivity and digestibility, and Yield was significantly negatively correlated with ADF. In the mixtures, D was also highly positively correlated with CP, EE, and RFV (*P*<0.001), and Yield did not show a significant negative correlation with CP or ADF (*P* > 0.05). In terms of weed resistance, in pure grass stands, total weed proportion (Weed) and grass weeds (GW) were highly significantly negatively correlated with Yield, D value, PFEU, and key nutritional metrics like CP and EE (*P*<0.01), whereas in the grass-legume mixtures, weed proportions showed no significant correlations with Yield, D value, or any nutritional parameters (*P* > 0.05). Regarding climatic influences, in pure grass stands, higher accumulated temperature (AT) was negatively correlated with Yield and D value (*P*<0.001). In the mixtures, precipitation (Pre) showed strong positive correlations with CP, EE, Ash, RFV, and FEU (*P*<0.001). Across both types of stands, Pre favored CP and Ash but decreased WSC, whereas sunshine hours (SH) significantly promoted WSC while suppressing CP and EE.

## 4 Discussion

### 4.1 Mechanisms underlying productivity dynamics and temporal complementarity

Pure grass stands and grass-legume mixtures demonstrated distinct productivity strategies. The superior total biomass accumulation of AM (9144.48 kg·ha^-1^, **Figure 3**) highlights the potential of HSG cultivars. The study by Lee et al. (2009) indicated that there is a positive correlation between the regrowth yield of perennial ryegrass and the WSC content of the stubble left after mowing. HSG accumulate elevated levels of WSC in the stele, which may serve as a readily available energy substrate for rapid regrowth following defoliation (Turner et al., 2007). However, relying solely on total annual yield masks critical seasonal instabilities. Pure grass stands experienced a sharp decline in productivity during the mid-to-late growing season (105–147 d, **Figure 2**). This phenomenon, often referred to as the “summer slump”, is typically attributed to the depletion of soil nitrogen availability and the physiological suppression of cool-season grasses under higher summer temperatures (Deng et al., 2020). In contrast, the grass-legume mixtures exhibited a strong compensatory growth effect. While ryegrass dominated the spring growth (21–84 d), WC thrived during the warmer summer months, becoming the dominant or co-dominant species from 126d onwards (**Figure 4**). This asynchronous growth pattern provides strong evidence for temporal niche differentiation (Nyfeler et al., 2009). WC has a higher optimal temperature for photosynthesis compared to perennial ryegrass and possesses the unique ability of biological nitrogen fixation (Davidson and Robson, 1986). As the competitive vigor of ryegrass waned due to seasonal stress, WC rapidly filled the available canopy gaps and utilized the light and thermal resources that the grass component could not efficiently exploit. Consequently, this interspecific complementarity enhanced yield stability. As shown in **Figure 3**, the mixtures maintained significantly lower temporal CV compared to pure stands. This finding aligns with the “insurance hypothesis” in biodiversity-ecosystem function theory, which suggests that functionally diverse communities are more resilient because the failure of one species is buffered by the success of another (Yachi and Loreau, 1999). Therefore, while pure grass stands like AM may offer maximum potential yield under ideal inputs, grass-legume mixtures provide a more stable and seasonally balanced forage supply, which is critical for grazing management in variable climates.

### 4.2 Nutritive value trade-offs: balancing energy and protein

A physiological trade-off between energy and protein content exists across treatments, as evidenced by the strong negative correlation between WSC and CP observed in this study (*P*<0.001) (**Figure 17**). Pure grass stands excelled in providing high-energy forage, maintaining elevated WSC content throughout the season. High WSC concentrations offer significant production advantages as they enhance the efficiency of ruminant digestion by providing readily available energy for rumen microbes to capture degradable protein, thereby reducing nitrogen excretion into the environment (Bertilsson et al., 2017). However, the energy advantage of pure grass stands came at the cost of protein limitations. The inclusion of WC fundamentally shifted this balance. While the mixtures exhibited lower WSC levels compared to pure grass stands due to the inherently low sugar content of legumes, they compensated with significantly higher CP concentrations. Crucially, the reduction in WSC in mixtures was offset by a favorable improvement in fiber characteristics. The grass-legume mixtures consistently recorded lower NDF values (32.57%–34.31%) compared to pure grass treatments (**Figure 6**). This morphological difference is likely due to the lower cell wall content in broad-leaved legumes compared to the fibrous stems of grasses (Guy et al., 2018). Lower NDF is a key determinant of voluntary feed intake; it is well established that decreasing NDF reduces the physical limitation on gut fill, thereby allowing animals to consume more dry matter (Allen et al., 2019). Therefore, the choice between these sowing modes represents a strategic nutritional decision. Pure grass stands offer a high-energy density forage that may require protein supplementation to balance the rumen C:N ratio (Reinsch et al., 2021). In contrast, grass-legume mixtures provide a more nutritionally balanced diet. By sacrificing some sugar concentration, the mixtures deliver a more optimized profile of high protein and low fiber, potentially supporting higher total nutrient intake without external supplementation.

### 4.3 Ecological stability and biological weed suppression

Beyond yield and quality, the sustainability of a grassland system is defined by its ability to maintain stable production and resist invasion over time. Our results demonstrate that increasing functional diversity through grass-legume mixtures significantly enhances system stability. **Figure 3** reveals that mixtures consistently buffered the seasonal fluctuations inherent in monocultures, driven by a complementary bimodal growth pattern where WC filled the ecological niche left by the cool-season grass during mid-summer. This phenomenon aligns with compensatory effects in ecology, where the presence of functionally distinct species ensures that if one species underperforms due to environmental stress, the other compensates, stabilizing the overall community biomass (Liang et al., 2022). Closely linked to this stability is the superior biological weed suppression observed in the mixtures. In the pure grass stands, the summer decline in PRG growth vigor left ecological niches vacant. This resource gap was rapidly exploited by weeds, with grass weed proportions reaching 12.91% in PR at 105d (**Figure 4**), which our correlation analysis showed was highly detrimental to total yield, D value, and key nutritional metrics like crude protein and ether extract (**Figure 17**). In contrast, the grass-legume mixtures exhibited strong biotic resistance, suppressing grass weeds to below 0.5% during the same period and showing no significant negative correlations between weed presence and forage quality or yield. The rapid expansion of WC during the mid-to-late growing season, which peaked at approximately 75% in the PR+WC treatment between 126 and 147 d, effectively closed the canopy, preempting the resources required for weed establishment. This mechanism of niche filling demonstrates that a spatially and temporally dense sward is the most effective defense against invasion (Mwangi et al., 2007). Results indicate that the internal stability of these mixtures is significantly influenced by cultivar characteristics. High-sugar grass cultivars, such as AberMagic, demonstrated superior competitive stability (PRG proportion CV of 42.06%) compared to the standard Premium variety (CV of 56.14%), preventing total legume dominance and maintaining a more balanced, synergistic sward structure under environmental fluctuations.

### 4.4 Comprehensive evaluation, agronomic strategy recommendations and limitation

To evaluate the comprehensive production potential of forage systems, one must break the limitations of single indicators and comprehensively consider FEU, PFEU, and their coupling coordination degree. Data indicate differences in the focus of different management models. Pure grass swards tend to maximize absolute output in the early growth stage, whereas legume-grass mixtures further optimize long-term seasonal stability and interspecific complementarity. According to the comprehensive performance of these indicators, the treatments can be divided into three tiers. The first tier consists of AM, which has an advantage in early-season yield maximization, and AA+WC, which possesses long-term system stability. The second tier includes Tri, AS, AA, AM+WC, and AS+WC, exhibiting intermediate overall performance. The third tier comprises Premium and PR+WC, characterized by large overall fluctuations and low productivity. For intensive systems prioritizing maximum yield, pure-sown AM represents a high-yield model. AM delivers the highest average PFEU and rapidly achieves a state of good coordination at 42 days, effectively combining high biomass with premium nutrition in the early growth stage. However, the pure-sown model has higher temporal variability and is prone to noticeable yield decline during the mid-growing season. Adopting this model requires a rational planning of the cutting schedule to mitigate the rapid decline in forage quality and counter the adverse effects of summer dormancy (Vinther, 2006). For long-term grazing systems demanding a reliable forage supply, the AA+WC mixture demonstrates a strong comprehensive advantage. The inclusion of white clover leverages ecological complementarity between species, significantly reducing temporal variability in productivity. AA+WC exhibits an exceptional late-season compensatory recovery capacity. At 147 days, it rebounds to intermediate coordination and maintains a high FEU, effectively extending the supply window of high-quality forage precisely when the yield and nutritional quality of pure grass swards experience severe incoordination. Other mixture combinations similarly provide reliable late-season performance, demonstrating that interspecific pairing successfully mitigates the traditional yield-quality trade-off. The consistently poor performance of the standard variety Premium reflects the necessity of utilizing improved traits. Premium exhibits weaker stability and plummets to moderate incoordination mid-season. Even when intercropped with legumes, the average PFEU of the combined system remains the lowest, indicating obvious incoordination between yield formation and nutrient accumulation. Building a highly coordinated and stable forage system requires not only the ecological benefits of species mixtures but also relies on improved traits (Edwards et al., 2007).

Finally, this field experiment was conducted during a single, exceptionally wet year (2223.8mm annual precipitation). These conditions likely favored white clover growth and mitigated the “summer slump.” While our results highlight the synergistic potential of combining improved traits with interspecific diversity, further multi-year validations under varying climatic conditions are warranted to confirm the generalizability of these management models.

## 6 Conclusions

This study highlights a trade-off between maximizing absolute productivity and ensuring seasonal resilience in temperate forage systems. Pure HSG monocultures maximize early-season dry matter yield and deliver highly energy-dense forage. But they remain highly susceptible to late-season yield declines and weed invasion. Integrating WC effectively buffers this “summer slump” through temporal niche complementarity. This functional diversity enhances long-term yield stability, provides robust biological weed suppression, and offers a more physiologically balanced ruminant diet by compensating for lower carbohydrate levels with boosted CP. Comprehensive evaluations utilizing the Coupling Coordination Degree, FEU, and PFEU indicate that management models must align with specific goals. Intensive systems demanding maximum total output benefit most from AberMagic. For long-term rotational grazing requiring a continuous feed supply, the AberAvon and white clover mixture is optimal, demonstrating exceptional late-season recovery and high coordination. Ultimately, the poor performance of standard control varieties emphasizes the absolute necessity of utilizing improved traits. Synergizing the ecological resilience of multi-species mixtures with the improved traits of HSG cultivars provides the optimal framework for sustainable, high-yielding agricultural systems.

## Data Availability

The raw data supporting the conclusions of this article will be made available by the authors, without undue reservation.
